# Unveiling the hidden diversity of neotropical *Steccherinum* and allied genera (*Steccherinaceae*, *Basidiomycota*)

**DOI:** 10.3897/imafungus.17.182915

**Published:** 2026-02-27

**Authors:** Mauro Carpes Westphalen, Nathalia Michele Martins Minosso, Nicolas do Carmo Regio, Adriana de Mello Gugliotta, Mario Rajchenberg, Rosa Mara Borges da Silveira

**Affiliations:** 1 Universidade Federal do Rio Grande do Sul, Instituto de Biociências, Programa de Pós-graduação em Botânica, Av. Bento Gonçalves 9500, Prédio 43.433, 91501-970, Porto Alegre, Rio Grande do Sul, Brazil CONICET and Centro de Investigación y Extensión Forestal Andino Patagónico, Área de Fitopatología y Microbiología Aplicada Esquel Argentina; 2 Universidade Federal de Pernambuco, Centro de Biociências (CB), Programa de Pós-Graduação em Biologia de Fungos, Av. da Engenharia, Cidade Universitária, 50670-420, Recife, PE, Brazil Universidade Federal do Rio Grande do Sul Porto Alegre Brazil; 3 Instituto de Pesquisas Ambientais, Núcleo de Conservação da Biodiversidade, Av. Miguel Estefano 3687, 04301-902, São Paulo, SP, Brazil Universidade Federal de Pernambuco Recife Brazil; 4 CONICET and Centro de Investigación y Extensión Forestal Andino Patagónico, Área de Fitopatología y Microbiología Aplicada, C.C. 14, 9200 Esquel, Chubut, Argentina Instituto de Pesquisas Ambientais, Núcleo de Conservação da Biodiversidade São Paulo Brazil

**Keywords:** Corticioid fungi, cryptic species, mating studies, multigene analysis, neotropical Funga, *
Polyporales
*

## Abstract

*Steccherinum* and its allied genera represent a morphologically complex group of fungi within the *Steccherinaceae*. In this study, we investigated, through morphological and multigene phylogenetic analyses, the diversity of odontioid/hydnoid *Steccherinum* s.l. collected in Brazil. Culture studies were conducted to compare mycelial morphology and growth rates among species, and mating tests were performed to assess sexual compatibility among related taxa. In addition, divergence-time estimates for the *Steccherinaceae* were generated using a concatenated five-gene dataset to contextualize the evolutionary history of the group. Molecular data revealed eight well-defined neotropical lineages in *Steccherinum*, including *S. larssonii*, *S. perparvulum*, *S. subochraceum*, and five new species: *S. bononiae*, *S. elegantissimum*, *S. molle*, *S. resinaceum*, and *S. undulatum*. The five newly described taxa are morphologically very similar and differ only in subtle diagnostic traits. Two additional new species were identified in *Cabalodontia*: *C. albofulva* and *C. brunnea*. Furthermore, the new combinations *C. lincangense* and *C. tenuissima* are proposed based on the phylogenetic data. Phylogenetic analyses also demonstrated that *S. perparvulum* comprises a species complex with three distinct lineages. Mating tests between two of these lineages showed a lack of sexual compatibility, indicating that they represent separate biological species that cannot be distinguished morphologically. Mycelial culture studies also revealed generally similar morphology with variable growth rates among taxa. Divergence-time estimates indicate a crown age of approximately 86.4 Ma for the *Steccherinaceae* and a predominantly Cenozoic diversification, with *Steccherinum* originating in the Eocene. Our findings highlight significant cryptic diversity within *Steccherinum* in the Neotropics and provide new insights into the taxonomy and phylogeny of the genus.

## Introduction

The genus *Steccherinum* Gray, typified by *S. ochraceum* (Pers.) Gray, is typically characterized by resupinate to effused-reflexed basidiomes with a hymenophore composed of pores, spines (or aculei), or, more rarely, a smooth surface. The basidiomes are generally thin and pale-colored, though some species may display brighter orange hues. A key feature distinguishing *Steccherinum* is its dimitic hyphal structure and the presence of thick-walled clavate skeletocystidia in the trama, often projecting into or above the hymenium. In most species, the cystidia are heavily encrusted, although in some cases they may be completely smooth. Overall, all species in the genus exhibit somewhat similar micromorphology, which contributes to the complexity of its taxonomy (Ryvarden 1991; Miettinen et al. 2012; Westphalen et al. 2021). Despite this, many new species have been described in *Steccherinum* in recent years (Westphalen et al. 2018; Liu and Dai 2021; Westphalen et al. 2021; Wu et al. 2021a; Dong et al. 2022; Dong et al. 2023; Liu et al. 2023; Wang et al. 2024), making it the largest genus in the *Steccherinaceae*, with over 120 associated names in the Index Fungorum database.

Morphologically, *Junghuhnia* Corda shares several similarities with *Steccherinum*, with the two genera traditionally distinguished by the presence of a poroid hymenophore in *Junghuhnia* and a hydnoid in *Steccherinum* (Ryvarden 1991). However, molecular studies have demonstrated that *Steccherinum* exhibits considerable variation in hymenophore morphology, encompassing species with both pores and spines (Miettinen et al. 2012; Miettinen and Ryvarden 2016; Westphalen et al. 2021). Consequently, the morphological distinction between the two genera has become less clear, and molecular data currently support the recognition of only two species in *Junghuhnia*: *J. crustacea* (Jungh.) Ryvarden and *J. pseudocrustacea* H.S. Yuan (Yuan et al. 2019). Additionally, *Cabalodontia* Piątek and *Etheirodon* Banker, both genera within the *Steccherinaceae*, also present somewhat similar morphological features to *Steccherinum*. However, *Cabalodontia* can be differentiated by its more fragile basidiomes, a monomitic hyphal structure, and cystidia that are typically broader at the base, tapering toward the apex. In contrast, *Etheirodon* is characterized by purplish basidiomes with strongly fimbriate margins, as well as smaller cystidia with thinner walls (Westphalen et al. 2021).

Although *Steccherinum* and its allied genera form a large group with numerous taxa distributed in temperate and tropical regions, their diversity in the Neotropics remains poorly explored, with very few studies dedicated to the group. To date, ten *Steccherinum* species have been described from the region, including six odontioid to hydnoid taxa (*S. basibadium* Banker, *S. diversum* Hjortstam & Melo, *S. filiferum* Yurchenko & K.H. Larss., *S. larssonii* Westphalen & Motato-Vásq., *S. perparvulum* Hjortstam & Ryvarden, and *S. subochraceum* Bononi & Hjortstam) (Hjortstam 1999; Westphalen et al. 2021; Yurchenko et al. 2023) and four poroid taxa (*S. amapaense* A.M.S. Soares & Ryvarden, *S. neonitidum* Westphalen & Tomšovský, *S. undigerum* (Berk. & M.A. Curtis) Westphalen & Tomšovský, and *S. polycystidiferum* (Rick) Westphalen, Tomšovský & Rajchenb.) (Hyde et al. 2017; Westphalen et al. 2018). In addition, one species has been described in *Etheirodon* (*E. purpureum* Westphalen) and another in *Cabalodontia* (*C. delicata* Westphalen & Motato-Vásquez) (Westphalen et al. 2021). Nonetheless, many *Steccherinum* specimens deposited in Brazilian herbaria are misidentified as *S. ochraceum*, a species originally described from northern Europe with a temperate distribution. To address this gap, the present study aimed to expand the knowledge of *Steccherinum* diversity in the Neotropics, with emphasis on odontioid/hydnoid taxa, through morphological examination of specimens, mycelial culture studies, and phylogenetic analyses, including divergence-time estimates for the *Steccherinaceae*. Our findings support the occurrence of several morphologically similar cryptic species of *Steccherinum* in the Neotropics. Seven new species are described and discussed, with key diagnostic features provided for their morphological differentiation.

## Materials and methods

### Morphological studies

The studied specimens were collected in southern and southeastern Brazil between 2017 and 2024. Additional collections from SP, ICN, HURM, PACA, K, and O (abbreviations according to Thiers continuously updated) were examined for morphological revision and comparison. Sections of basidiomes were observed under a compound microscope. Cotton blue (Merck 1275) in a lactic acid solution were used to assess cyanophilic reactions of spores and hyphae (denoted as CB+ or CB−) and to measure microstructures. Since all species in the *Steccherinaceae* are negative in Melzer’s reagent, these reactions are not shown in the descriptions, although they were tested. A minimum of 25 measurements of each structure was taken when possible. Illustrations of the cystidia and basidiospores were prepared using a drawing tube at 1000× magnification. Measurement abbreviations and codes used are as follows: Lm × Wm = mean length and width; Q = range of length/width ratio; Qm = mean length/width ratio; and n = x/y (where x = number of measurements from y specimens). Numbers in parentheses indicate extreme sizes observed in less than 5% of the measurements.

### DNA extraction and PCR amplification

DNA was extracted from either fungal cultures or small sections of dried basidiomes using a lysis buffer containing 2% CTAB, 1.4 M NaCl, 0.10 M Tris-HCl, and 20 mM EDTA, incubated at 65 °C for a minimum of 2 hours. After a chloroform extraction step, DNA was precipitated with isopropyl alcohol (Doyle 1987). PCR amplification of the ITS1-5.8S-ITS2 rDNA (ITS), 28S rDNA (LSU), and mtSSU regions was performed using primers ITS1/ITS4, LR0R/LR7, and MS1/MS2, respectively (Nikolcheva and Bärlocher 2004), following the methods outlined by Tomšovský et al. (2010). Amplification of the translation elongation factor 1-α (*tef*1-α) gene was carried out using the primers 983F/2218R or 983F/1567R (Matheny et al. 2007). The region spanning domains A and C of the largest RNA polymerase II subunit (*rpb*1) was amplified using primers rpb1-Af/rpb1-Cr (Matheny et al. 2002). For *tef*1-α and *rpb*1, a touchdown PCR protocol was used, in which the annealing temperature gradually decreased from 60 °C to 50 °C. After amplification, the products were purified with the ExoSAP-IT enzyme (Thermo Fisher), following the manufacturer’s protocols. Sequencing was performed by MacroGen Inc. (Seoul, South Korea), using the same primers as those employed in PCR amplification.

### Culture studies

Spore prints from freshly collected specimens were obtained and used to prepare both monosporic and polysporic cultures. The cultures were grown on Malt Extract Agar (MEA) or Potato Dextrose Agar (PDA) at 25 °C. Intraspecimen mating system tests were conducted following Hallenberg (1984), using ten monosporic cultures in each confrontation. After identification of the mating types, monosporic culture confrontations among closely related taxa were carried out according to Hallenberg (1984). For mycelial growth rate analysis and morphological observation and comparison, culture preparation was adapted from the methods outlined by Nobles (1965). To this end, inocula measuring approximately four millimeters of mycelium were placed at the edge of 9 cm diameter plates containing MEA. For each specimen, five plates were prepared and incubated at 25 °C for six weeks. Weekly, the following characteristics were examined: 1) macromorphology of the mycelium; 2) growth rate expressed in millimeters; and 3) micromorphology of the inner and advancing zones of the mycelium. Growth rate measurements were performed in triplicate for each specimen, with each plate measured from the inoculum endpoint to the edge of the mycelium.

### Phylogenetic analyses

Phylogenetic analyses were conducted using two datasets: one focused on the genus *Steccherinum* and another on *Cabalodontia*. The *Steccherinum* dataset included ITS, 28S, and *tef*1-α markers and encompassed all species with sequence data currently available. The *Cabalodontia* dataset included five molecular markers (ITS, 28S, *tef*1-α, *rpb*1, and mtSSU). Reference sequences were selected from Miettinen et al. (2012), Justo et al. (2017), and Westphalen et al. (2021), together with additional sequences retrieved through BLAST searches in the NCBI database. A summary of the sequences used in this study is provided in Table 1.

**Table 1.** List of sequences used in this study.

Specimen	Voucher	Loc.	GenBank No.					References
			ITS	28S	*tef1-α*	*rbp1*	mtSSU	
*Agaricus campestris*	LAPAG370	-	KM657927	KR006607	KR006636			Zhou et al. (2016)
*Alloclavaria purpurea*	Miettinen 18831	US	ON188807	ON188807	OQ776787	OQ776825	ON228494	Viner et al. 2024
*Antella americana*	HHB-4100-Sp	US	KP135316	KP135196		KP134885		Floudas and Hibbett 2015
*Antella chinensis*	Dai 9019	CN	JX110844	KC485542				Yuan 2013
*Antrodiella faginea*	KHL 11977	NO	JN710514	JN710514	JN710712		JN710658	Miettinen et al. 2012
*Antrodiella micra*	CLZhao 10185	CN	MZ713651	MZ713834	OK000964	OK000925	MZ958847	Wu and Zhao (unpub.)
*Antrodiella stipitata*	FD–136	US	KP135314	KP135197		KP134886		Floudas and Hibbett 2015
*Antrodiella trivialis*	MCW 369/12	BR	MH475302	MH475302	MH475314			Westphalen et al. 2019
*Aphanobasidium pseudotsugae*	CFMR:HHB-822	US	GU187509	GU187567	GU187695	GU187455		Binder et al. 2010
*Atraporiella neotropica*	Ryvarden 4447	BZ	HQ659221	HQ659221				Miettinen and Rajchenberg 2012
*Atraporiella yunnanensis*	CLZhao 604	CN	MF962482	MF962485	OK000966		MZ958849	Wu et al. 2017
*Austeria citrea*	X1171	NZ	JN710511	JN710511				Miettinen et al. 2012
*Butyrea luteoalba*	FP–105786	US	KP135320	KP135226		KP134887		Floudas and Hibbett 2015
*Butyrea luteoalba*	KHL 13238b	EE	JN710558	JN710558	JN710719		JN710682	Miettinen et al. 2012
*Butyrea japonica*	MN 1065	JP	JN710556	JN710556	JN710718		JN710680	Miettinen et al. 2012
*Cabalodontia albofibrillosa*	CLZhao 5032	CN	MW204589	MW204578	OK000982	OK000935	MZ958886	Wu et al. 2021a
*Cabalodontia albofibrillosa*	CLZhao 5024	CN	MW204587	MW204576	OK000980	OK000933	MZ958884	Wu et al. 2021a
*Cabalodontia albofibrillosa*	CLZhao 8722	CN	MZ713669	MZ713811				Wu and Zhao (unpub.)
*Cabalodontia albofibrillosa*	SWFC 006394	CN	MK894083					Zhao (unpub.)
*Cabalodontia albofibrillosa*	Sanyal 6903	IN	KP401770					Sanyal (unpub.)
*Cabalodontia albofulva*	MCW 563/17	BR	PX523825	PX523825	PX289988	PX289990		This study
*Cabalodontia brunnea*	MCW 600/18	BR	PX523826	PX523826	PX289989			This study
*Cabalodontia delicata*	MCW 693/19	BR	MT849297	MT849297	MT833936	MT833948		Westphalen et al. 2021
*Cabalodontia delicata*	MCW 564/17	BR	MT849295	MT849295	MT833934	MT833947	PX512156	Westphalen et al. 2021
*Cabalodontia lincangense*	Zhao24988	CN	OR096196	OR461455	OR541930	OR683157	OR469126	Dong et al. 2024
* Cabalodontia queletii *	FCUG 722	SE	AF141626					Hallenberg and Parmasto (unpub.)
*Cabalodontia queletii*	CBS: 233.56	FR	MH857599	MH869147				Vu et al. 2019
*Cabalodontia tenuissima*	CLZhao 5100	CN	MW204584	MW204573			MZ958882	Wu et al. 2021a
*Cabalodontia tenuissima*	CLZhao 3153	CN	MW204582	MW204571		OK000932	MZ958881	Wu et al. 2021a
*Callistosporium graminicolor*	AFTOL-ID 978	US	DQ484065	AY745702	GU187761	GU187493		Binder et al. 2010
*Cerrena unioclor*	KHL-GB	SE	JQ031127	JQ031127	JX109891		JN710663	Miettinen et al. 2012
*Citripora afrocitrina*	X596	UG	JN710508	JN710508	JN710710		JN710655	Miettinen et al. 2012
*Citripora bannaensis*	X243	CN	JN710526	JN710526				Miettinen et al. 2012
*Coniferiporia weirii*	FP-135667-SP	US	MT420702	MT416459	MT470386	MT376002	MT386058	Wang et al. 2022
*Cotylidia* sp.	AFTOL-ID 700	US	AY854079	AY629317	AY885148	AY864868		Wang et al. (unpub.)
*Etheirodon fimbriatum*	HR97926	CZ	MT849299		MT833937	MT833954		Westphalen et al. 2021
*Etheirodon fimbriatum*	HR98811	CZ	MT849300		MT833938	MT833955		Westphalen et al. 2021
*Etheirodon aff. fimbriatum*	FP-102075	US	KY948821	KY948864		KY948950	AF518695	Justo et al. 2017
*Etheirodon purpureum*	MCW 642/18	BR	MT849301	MT849301	MT833939			Westphalen et al. 2021
*Etheirodon roseoalbum*	CLZhao 24770	CN	OR096187	OR541929	OR541929	OR683155	OR469121	Dong et al. 2024
*Flabellophora parva*	21	BR	OR098554	OR098556				Saragiotto (unpub.)
*Flabellophora* sp. 3	X1277	ID	JN710535	JN710535			JN710669	Miettinen et al. 2012
*Flaviporus brownii*	MCW362/12	BR	KY175008	KY175008	KY175022			Westphalen et al. 2018
*Flaviporus lacteus*	MCW654/18	BR	OL638488	OL638488	OL631200			Westphalen et al. 2022
*Flaviporus cf. subglobisporus*	X1092	BR	JN710542	JN710542			JN710671	Miettinen et al. 2012
*Frantisekia fissiliformis*	CBS 435.72	US	MH860521	MH872232				Vu et al. 2019
*Frantisekia mentschulensis*	BRNM 710170	CZ	FJ496670	FJ496728			FJ496748	Tomšovský et al. 2010
*Irpex oreophilus*	X214	FI	JN710548	JN710548				Miettinen et al. 2012
*Irpex oreophilus*	HHB13202sp	US	KY948824			KY948949		Justo et al. 2017
*Junghuhnia crustacea*	CLZhao 11926	CN	MZ713655	MZ713838		OK000927	MZ958854	Wu and Zhao (unpub.)
*Junghuhnia pseudocrustacea*	Yuan 6160	CN	MF139552					Yuan et al. 2019
*Junghuhnia pseudocrustacea*	Zhou 283	CN	MF139551					Yuan et al. 2019
*Kneiffiella alutacea*	Miettinen 21701	FI	ON188808	ON188808	OQ776802	OQ776841	ON228491	Viner et al. 2024
*Lachnella villosa*	AFTOL-ID 525	NL	DQ097362	DQ097362	GU187721		DQ097381	Binder et al. 2006
*Lamelloporus americanus*	X670	EC	JN710567	JN710567				Miettinen et al. 2012
*Loweomyces fractipes*	X1253	US	JN710569	JN710569			JN710689	Miettinen et al. 2012
*Loweomyces tomentosus*	MCW366/12	BR	KX378870	KX378870				Westphalen et al. 2016
*Metuloidea reniforme*	MCW 542/17	BR	MT849303	MT849303	MT833940	MT833950		Westphalen et al. 2021
*Metuloidea rhinocephala*	X460	AU	JN710562	JN710562			JN710686	Miettinen et al. 2012
*Mycorrhaphium adustum*	8024	US	JN710573	JN710573	JN710727		JN710692	Miettinen et al. 2012
*Mycorrhaphium hispidum*	MCW429/13	BR	MH475307	MH475307	MH475318			Westphalen et al. 2019
*Mycorrhaphium subadustum*	Yuan 12976	CN	MW491378	MW488040				Cao et al. 2021
*Nigroporus austroasianus*	Dai 28512	CN	PQ327581	PQ327583	PQ540992			Li et al. 2025b
*Nigroporus vinosus*	8182	US	JN710575	JN710575	JN710728		JN710693	Miettinen et al. 2012
*Peniophorella praetermissa*	AFTOL-ID 518	-	AY854081	AY700185	AY885150	AY864871		Nilsson et al. (unpub.)
*Schizophyllum radiatum*	AFTOL-ID 516	PA	AY571060	AY571023		DQ447939	DQ097383	Matheny et al. 2006
*Steccherinum amapaense*	M245	BR	KY977406	KY977405				Hyde et al. 2017
*Steccherinum autumnale*	Spirin 2957	RU	JN710549	JN710549	JN710716		JN710675	Miettinen et al. 2012
*Steccherinum austrosinense*	Dai 17540	CN	MN871755	MN877768				Du et al. 2020
*Steccherinum austrosinense*	Dai 17540	CN	MN871756	MN877769				Du et al. 2020
*Steccherinum bononiae*	AG 1615	BR	PV434851	PV434851				This study
*Steccherinum bononiae*	MCW 547/17	BR	PV434850	PV434850	PV442019			This study
*Steccherinum bononiae*	MCW 557/17	BR	PV434854	PV434854	PV442020	PV439912		This study
*Steccherinum bononiae*	MCW 726/22	BR	PV434852	PV434852		PV439913		This study
*Steccherinum bononiae*	NR71	BR	PV434853	PV434853				This study
*Steccherinum bononiae*	MV446	BR	PV434855					This study
*Steccherinum bourdotii*	HR 99893	CZ	MT849311		MT833945	MT833951		Westphalen et al. 2021
*Steccherinum bourdotii*	MT 10/19	CZ	MT849312		MT833944	MT833952		Westphalen et al. 2021
*Steccherinum collabens*	KHL 11848	SE	JN710552	JN710552	JN710717		JN710677	Miettinen et al. 2012
*Steccherinum elegantissimum*	MCW 633/18	BR	PV434856	PV434856	PV442016	PV439914		This study
*Steccherinum elegantissimum*	MCW 720/21	BR	PV434857	PV434857	PV442017			This study
*Steccherinum elegantissimum*	MCW 721/21	BR	PV434858		PV442018	PV439915		This study
*Steccherinum filiferum*	M-5230	EC	OP279612					Yurchenko et al. 2023
*Steccherinum fimbriatellum*	Miettinen 2091	RU	JN710555	JN710555				Miettinen et al. 2012
*Steccherinum formosanum*	Dai 19345	CN	MN871759	MN877772				Du et al. 2020
*Steccherinum formosanum*	TFRI 652	–	EU232184	EU232268				Chou et al. (unpub.)
*Steccherinum hirsutum*	CLZhao 4222	CN	MW290040	MW290054	OK000973	OK000931	MZ958871	Dong et al. 2022
*Steccherinum incrustans*	Dai 19442	CN	ON182084	ON182087				Liu et al. 2023
*Steccherinum juniperi*	Dai 23931	CN	OP956077	OP956031				Liu et al. 2023
*Steccherinum lacerum*	Niemelä 8246	FI	JN710557	JN710557				Miettinen et al. 2012
*Steccherinum laeticolor*	Fp102480sp	US	KY948823	KY948868.1		KY948948		Justo et al. 2017
*Steccherinum larssonii*	MCW 593/17	BR	MT849306	MT849306	MT833941	MT833956		Westphalen et al. 2021
*Steccherinum larssonii*	MCW 594/17	BR	MT849307	MT849307	MT833942			Westphalen et al. 2021
*Steccherinum laxum*	KHL 12268	US	JN710577	JN710577	JN710729		JN710694	Miettinen et al. 2012
*Steccherinum meridionale*	MR 284	AR	KY174992	KY174992	KY175019			Westphalen et al. 2018
*Steccherinum molle*	MCW 568/17	BR	PV434863	PV434863	PV442021			This study
*Steccherinum molle*	MCW 641/18	BR	PV434864	PV434864	PV442022			This study
*Steccherinum molle*	MCW 661/18	BR	PV434865	PV434865				This study
*Steccherinum molle*	MCW 687/19	BR	PV434866	PV434866	PV442023			This study
*Steccherinum molle*	MCW 734/22	BR	PV434867		PV442024	PV439916		This study
*Steccherinum molle*	MCW 739/23	BR	PV434868		PV442025	PV439917		This study
*Steccherinum molle*	MV450	BR	PV434869					This study
*Steccherinum nandinae*	Dai 21107	CN	MN833677	MN833679				Du et al. 2020
*Steccherinum neonitidum*	MCW 371/12	BR	KY174990	KY174990	KY175017			Westphalen et al. 2018
*Steccherinum nitidum*	KHL 11903	SE	JN710560	JN710560	JN710721		JN710684	Miettinen et al. 2012
*Steccherinum aff. nitidum*	FP-105195-Sp	US	KP135323	KP135227		KP134888		Floudas and Hibbett 2015
*Steccherinum ochraceum*	KHL11902	SE	JN710590	JN710590	JN710730		JN710700	Miettinen et al. 2012
*Steccherinum perparvulum*	524/17	BR	PV434837	PV434837	PV442009	PV439905		This study
*Steccherinum perparvulum*	592/17	BR	PV434847	PV434847				This study
*Steccherinum perparvulum*	659/18	BR	PV434845	PV434845	PV442006	PV439906		This study
*Steccherinum perparvulum*	692/19	BR	PV434838	PV434838	PV442008			This study
*Steccherinum perparvulum*	543/17	BR	PV434839	PV434839	PV442010			This study
*Steccherinum perparvulum*	710/20	BR	PV434841		PV442011		PX693402	This study
*Steccherinum perparvulum*	742/23	BR	PV434846		PV442007	PV439907	PX693403	This study
*Steccherinum perparvulum*	744/23	BR	PV434843		PV442013	PV439908		This study
*Steccherinum perparvulum*	MV728	BR	PV434840					This study
*Steccherinum perparvulum*	MV815	BR	PV434844	PV434844	PV442005			This study
*Steccherinum perparvulum*	NR186	BR	PV434842		PV442012			This study
*Steccherinum polycystidiferum*	MCW 419/12	BR	KY174995	KY174995	KY175021			Westphalen et al. 2018
*Steccherinum pseudozilingianum*	Kulju 1004	FI	JN710561	JN710561	JN710722		JN710685	Miettinen et al. 2012
*Steccherinum puerense*	Miettinen 13705	ID	JN710592	JN710592	JN710731		JN710701	Miettinen et al. 2012
*Steccherinum puerense*	CLZhao 3122	CN	MW682341		OK000976			Wu et al. 2021b
*Steccherinum resinaceum*	MCW 540/17	BR	PV434870	PV434870				This study
*Steccherinum resinaceum*	MCW 551/17	BR	PV434871	PV434871	PV442030	PV439918		This study
*Steccherinum resinaceum*	MCW 665/19	BR	PV434872	PV434872	PV442031			This study
*Steccherinum resinaceum*	MCW 679/19	BR	PV434873	PV434873	PV442032			This study
*Steccherinum robustius*	1195	SE	JN710591	JN710591				Miettinen et al. 2012
*Steccherinum rubigimaculatum*	CLZhao 10638	CN	MW682344	MW682340	OK000977			Wu et al. 2021b
*Steccherinum rubigimaculatum*	CLZhao 4069	CN	MW682343	MW682339				Wu et al. 2021b
*Steccherinum subochraceum*	730/22	BR	PV434859		PV442026			This study
*Steccherinum subochraceum*	746/23	BR	PV434861	PV434861	PV442027	PV439909		This study
*Steccherinum subochraceum*	748/23	BR	PV434860	PV434860	PV442028	PV439910		This study
*Steccherinum subochraceum*	761/24	BR	PV434862	PV434862	PV442029	PV439911		This study
*Steccherinum subtropicum*	CLZhao F11059	CN	OP799390	OP799377				Dong et al. 2023
*Steccherinum tenue*	KHL 12316	US	JN710598	JN710598	JN710733		JN710705	Miettinen et al. 2012
*Steccherinum tenuispinum*	LE231603	RU	KM411452	KM411469	KM411484			Zmitrovich and Kovalenko 2016
*Steccherinum undigerum*	MCW 436/13	BR	KY174988	KY174988	KY175020			Westphalen et al. 2018
*Steccherinum undulatum*	MCW 743/23	BR	PV434848	PV434848	PV442014			This study
*Steccherinum undulatum*	MCW 760/24	BR	PV434849	PV434849	PV442015	PV439919		This study
*Steccherinum wumengshanense*	CLZhao 23586	CN	OR658995	OR999392				Wang et al. 2024
*Steccherinum yunnanense*	CLZhao 1445	CN	MW290042	MW290056	OK000984		MZ958889	Dong et al. 2022
*Steccherinum* sp.	FD-26	US	KP135322	KP135289		KP134889		Floudas and Hibbett 2015
*Steccherinum* sp. 2	Miettinen 9300	ID	JN710593	JN710593				Miettinen et al. 2012
*Steccherinum* sp. 3	Miettinen 14391	ID	JN710594	JN710594	JN710732			Miettinen et al. 2012
*Steccherinum* sp. 4	Miettinen 13755	ID	JN710596	JN710596				Miettinen et al. 2012
*Trullella duracina*	MCW410/12	BR	MH475309	MH475309				Westphalen et al. 2019
*Trullella polyporoides*	X510	VE	JN710602	JN710602				Miettinen et al. 2012

Sequence alignments were performed using MAFFT 7 online (http://mafft.cbrc.jp/alignment/server/) under the auto mode strategy. *tef*1-α introns were excluded from the analyses. The *Steccherinum* dataset was divided into three partitions: ITS, 28S, and *tef*1-α. The *Cabalodontia* dataset was divided into six partitions: ITS, 28S, *tef*1-α, *rpb*1, *rpb*1 introns, and mtSSU. Bayesian inference (BI) analyses were carried out in MrBayes 3.2.6 (Ronquist et al. 2012), with substitution models selected for each partition based on AICc values computed in jModelTest 2.1.4 (Darriba et al. 2012). The selected models were GTR + I + G for ITS and *rpb*1, TIM3 + I + G for 28S, TIM2 + I + G for *tef*1-α, and TPM2uf + I + G for *rpb*1 introns. The proportion of invariable sites (I) and gamma-distributed rates (G) were set according to the models selected for each partition. Four independent MCMC chains were run for 10 million generations, sampling every 1000 generations. The first 25% of trees were discarded as burn-in, and the remaining trees were used to generate a 50% majority-rule consensus tree. Posterior probabilities greater than 0.9 were considered strongly supported and values above 0.8 moderately supported. The Maximum Likelihood (ML) analysis was conducted in RAxML-HPC 8 (Stamatakis 2014) using a rapid bootstrap analysis and a search for the best-scoring ML tree. The same partitioning scheme used for BI was adopted, applying the GTRGAMMA model. Bootstrap values above 80% were considered statistically significant. All analyses were performed through the CIPRES Science Gateway portal (Miller et al. 2011).

### Divergence time estimation

Divergence times were estimated using BEAST v2.7.7 (Bouckaert et al. 2019) with a five-gene dataset composed of ITS + 28S + *tef*1-α + *rpb*1 + mtSSU sequences aligned with MAFFT 7 online (Katoh and Standley 2013) under the auto mode strategy. *Archaeomarasmius leggetti* Hibbett, D. Grimaldi & Donoghue (Hibbett et al. 1997) and *Quatsinoporites cranhamii* S.Y. Sm., Currah & Stockey (Smith et al. 2004) were used as secondary fossil calibrations for *Agaricales* and *Hymenochaetales*, respectively. Six partitions were selected (ITS, 28S, *tef*1-α, *rpb*1, *rpb*1 introns, and mtSSU), and GTR + G was evaluated as the best-fit evolutionary model for the *rpb*1 introns partition and GTR + I + G for the other five partitions in jModelTest using the Corrected Akaike Information Criterion (AICc) (Darriba et al. 2012). Detailed parameters and fossil calibrations followed those used by Wang et al. (2023) and Li et al. (2025a) when generating the XML file in BEAUti v2. Two independent analyses of 100 million generations, sampling every 10,000 generations, were performed. Chain convergence was evaluated in Tracer v1.7.1 (Rambaut et al. 2018), and the two runs were combined, discarding 20% of states from each as burn-in, in LogCombiner v2.7.7 (Bouckaert et al. 2019), rendering a file with 8000 trees. A Maximum Clade Credibility (MCC) tree was then summarized, annotating clades with ≥ 0.8 posterior probability, in TreeAnnotator v2.7.7 (Bouckaert et al. 2019). The resulting tree was visualized in FigTree v1.4.4 (http://tree.bio.ed.ac.uk/software/figtree/) to obtain the mean ages and 95% Highest Posterior Density (HPD) values (Drummond and Rambaut 2007). A 95% HPD marks the shortest interval that contains 95% of the sampled values. Since the main focus of this study was on *Steccherinum* and its related genera, taxa of *Steccherinaceae* with sequence data for at least three molecular markers were prioritized. However, for genera represented by only two available markers, those data were nevertheless included to ensure adequate representation in the analyses.

## Results

**Figure 1.**
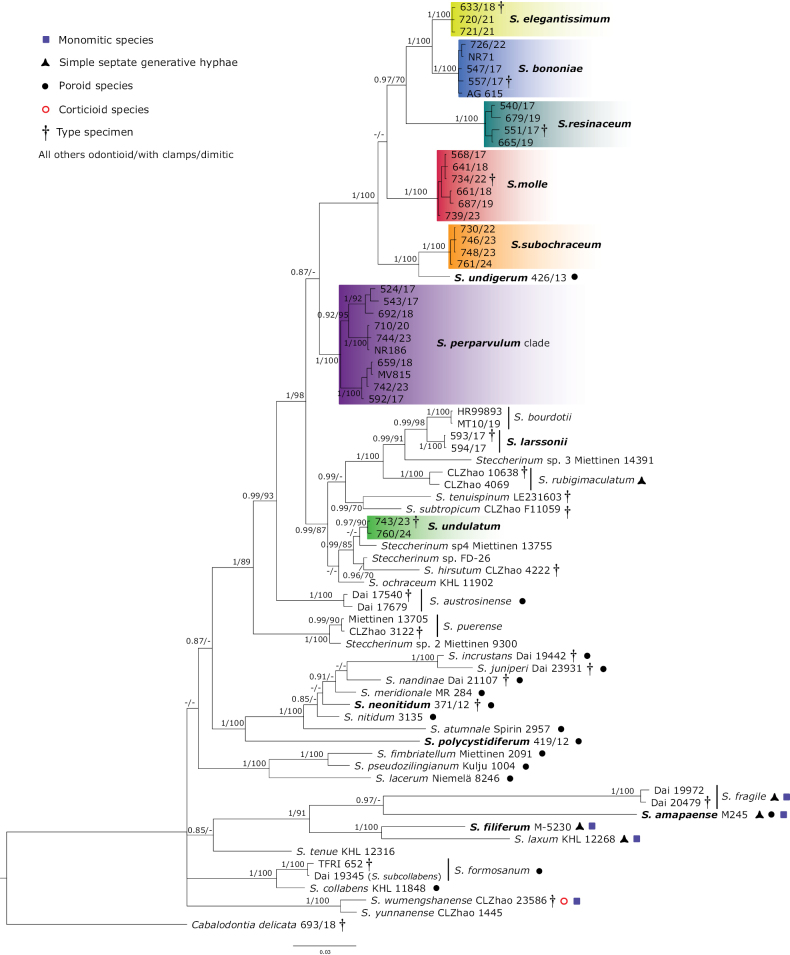
*Steccherinum* phylogenetic tree of ITS–28S–*tef*1-α regions conducted by Bayesian analysis (for legends and numbers, see Table 1). Numbers at branches indicate Bayesian posterior probability and maximum likelihood bootstrap values. The scale bar indicates the number of expected substitutions per position. Neotropical taxa are highlighted in bold. Species with newly obtained sequence data are highlighted in colors. Type voucher specimens are indicated with a †.

**Figure 2.**
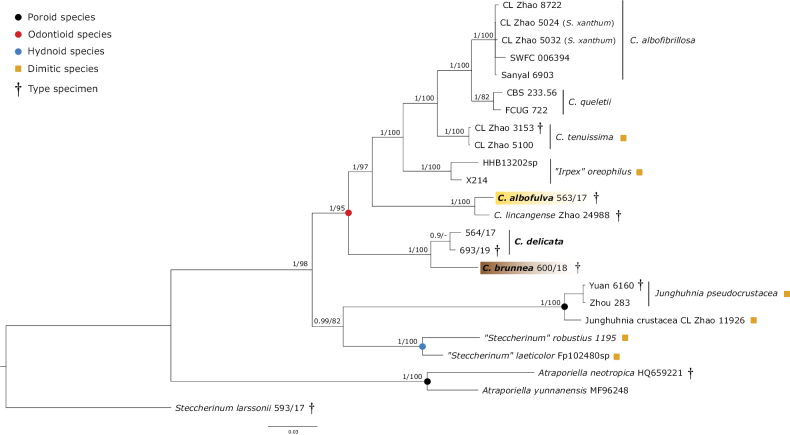
*Cabalodontia* phylogenetic tree of ITS–28S–*tef*1-α–*rpb*1–mtSSU regions conducted by Bayesian analysis (for legends and numbers, see Table 1). Numbers at branches indicate Bayesian posterior probability and maximum likelihood bootstrap values. The scale bar indicates the number of expected substitutions per position. Neotropical taxa are highlighted in bold. New species are highlighted in colors. Type voucher specimens are indicated with a †.

The newly obtained molecular data revealed five new lineages in *Steccherinum*, represented by the new species *S. bononiae*, *S. elegantissimum*, *S. molle*, *S. resinaceum*, and *S. undulatum* (Fig. 1), and two new species in *Cabalodontia*: *C. albofulva* and *C. brunnea* (Fig. 2). Sequence data for *S. perparvulum* and *S. subochraceum* are presented here for the first time and confirm them as distinct lineages in *Steccherinum* (Fig. 1). Furthermore, *S. lincangense* and *S. tenuissimum* are nested within *Cabalodontia* and are combined into that genus.

In our phylogenetic analyses, *S. xanthum* nested in a single lineage with *C. albofibrillosa* (Fig. 1). The ITS sequences of both species, including the paratypes of *S. xanthum* (CLZhao 5032 and CLZhao 5024), are nearly identical, differing by only about two base pairs. Therefore, we consider them conspecific, with the older name *C. albofibrillosa* taking priority. The same applies to *S. subcollabens*, whose type material (Dai 19345) presents ITS sequences identical to the type of *S. formosanum* (TFRI 652) and represents a synonym of the latter (Fig. 1).

**Figure 3.**
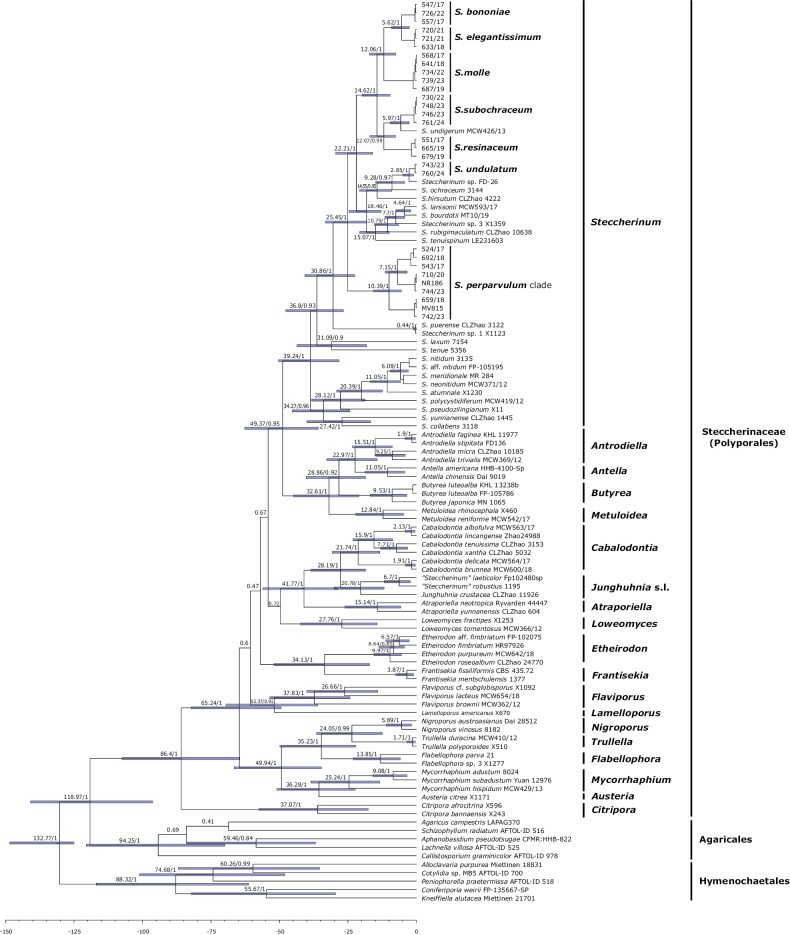
Maximum Clade Credibility (MCC) tree with divergence time estimations of *Steccherinaceae* inferred from Bayesian evolutionary analysis based on an ITS–28S–*tef*1-α–*rpb*1–mtSSU dataset. Mean ages of the nodes with at least 0.8 posterior probability were annotated along with the 95% highest posterior densities, which are marked by horizontal bars. Divergence time values and the scale bar indicate millions of years (Myr).

Notably, *S. perparvulum* encompasses three distinct lineages (Figs 1, 3)exhibiting variations in the ITS and *tef*1-α regions. However, no clear morphological differences or culture variation regarding morphology or growth rates were observed to distinguish these lineages. Mating tests conducted further corroborated that the observed genetic differences represent separate biological species. Consequently, we designated this group as the *S. perparvulum* species complex for now (see the Taxonomy section for further comments).

To compose the divergence-time estimation, we used a concatenated five-gene dataset, including 107 specimens, of which 97 belong to 19 different genera of the *Steccherinaceae*. Ten additional taxa from *Agaricales* and *Hymenochaetales* were used to represent the genetic diversity of the two secondary fossil calibrations used in the analysis. The MCC tree recovered (Fig. 3) estimates the ancestor of the *Steccherinaceae* with a stem age of approximately 118.97 Myr (95% height = 140.89–96.26 Myr) and a crown age of 86.4 Myr (95% height = 107.49–67.49 Myr), emerging in the early to late Cretaceous period. Estimated stem ages for its genera range from 86.4 Myr (95% height = 107.49–64.89 Myr) for *Citripora* Miettinen to 22.97 Myr (95% height = 32.76–14.39 Myr) for the *Antrodiella/Antella* clade. The genus *Steccherinum* was recovered with a mean stem age of 49.37 Myr (95% height = 62.69–35.79 Myr) and a mean crown age of 39.24 Myr (95% height = 50.4–28.27 Myr), placing its origin in the Eocene. The estimated ages for its species range from 31.09 Myr (95% height = 43.66–18.15 Myr) to 0.44 Myr (95% height = 1.07–0.02 Myr). On the other hand, *Cabalodontia* was recovered with a mean stem age of 28.19 Myr (95% height = 38.57–18.59 Myr) and a mean crown age of 21.74 Myr (95% height = 30.76–13.37 Myr), placing its origin in the Oligocene. The estimated ages for its species range from 7.1 Myr (95% height = 13.05–3.16 Myr) to 1.91 Myr (95% height = 3.74–0.53 Myr). Table 2 summarizes the estimated divergence times of the main nodes of the *Steccherinaceae* with at least 0.8 posterior probability. The international chronostratigraphic chart follows Cohen et al. (2013; updated) (URL: http://www.stratigraphy.org/ICSchart/ChronostratChart2022-10.pdf).

**Table 2.** Estimated divergence times of main nodes of the Steccherinaceae. PP stands for “Posterior Probabilities”. Nodes with PP < 0.8 were not annotated.

Node	Mean of stem age / 95% HPD (Mya)	PP	Mean of crown age / 95% HPD (Mya)	PP	Period
* Steccherinaceae *	118.97 (140.89–96.26)	1	86.4 (107.49–67.49)	1	Late Cretaceous
* Steccherinum *	49.37 (62.69–35.79)	0.95	39.24 (50.4–28.27)	1	Eocene
* Cabalodontia *	28.19 (38.57–18.59)	1	21.74 (30.76–13.37)	1	Oligocene
“*Antrodiella*” clade	49.37 (62.69–35.79)	0.95	32.61 (44.97–20.95)	1	Eocene
“*Junghuhnia*” clade	41.77 (56.13–28.76)	1	28.19 (38.57–18.59)	1	Eocene
* Loweomyces *	-	-	27.76 (42.34–14.4)	1	Oligocene
*Etheirodon/Frantisekia*	-	-	34.13 (52.0–17.04)	1	Eocene
*Flaviporus/Lamelloporus*	-	-	52.37 (69.56–36.15)	0.91	Eocene
“*Mycorrhaphium*” clade	65.24 (82.26–49.37)	1	49.94 (66.53–34.63)	1	Paleocene
* Citripora *	86.4 (107.49–64.89)	1	37.07 (57.52–17.59)	1	Eocene

**Figure 4.**
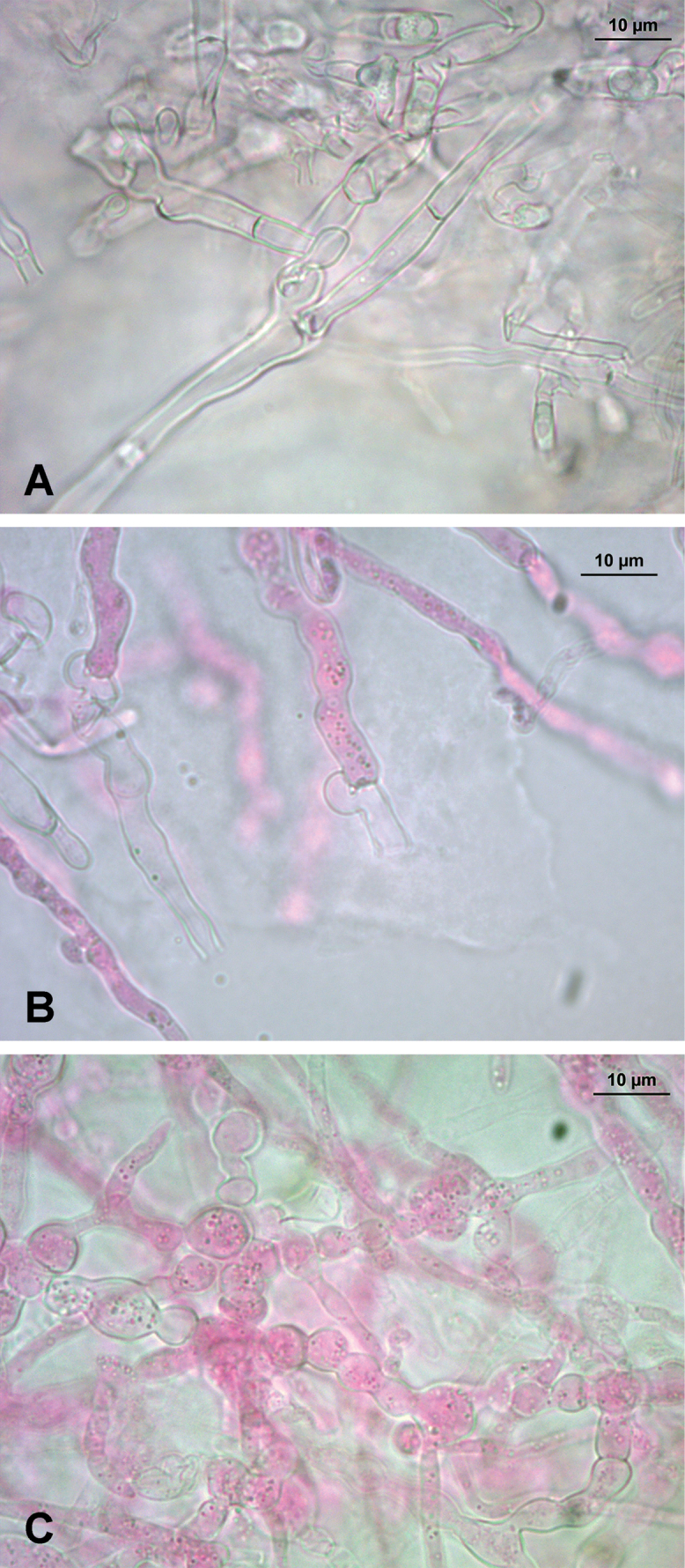
Microscopic features of *Steccherinum* spp. cultures. **A** generative hyphae with simple septa in strain 748/23 (*S. subochraceum*) **B** generative hyphae with clamps in strain 761/24 (*S. subochraceum*) **C** intercalary chlamydospores in strain 665/19 (*S. resinaceum*).

**Figure 5.**
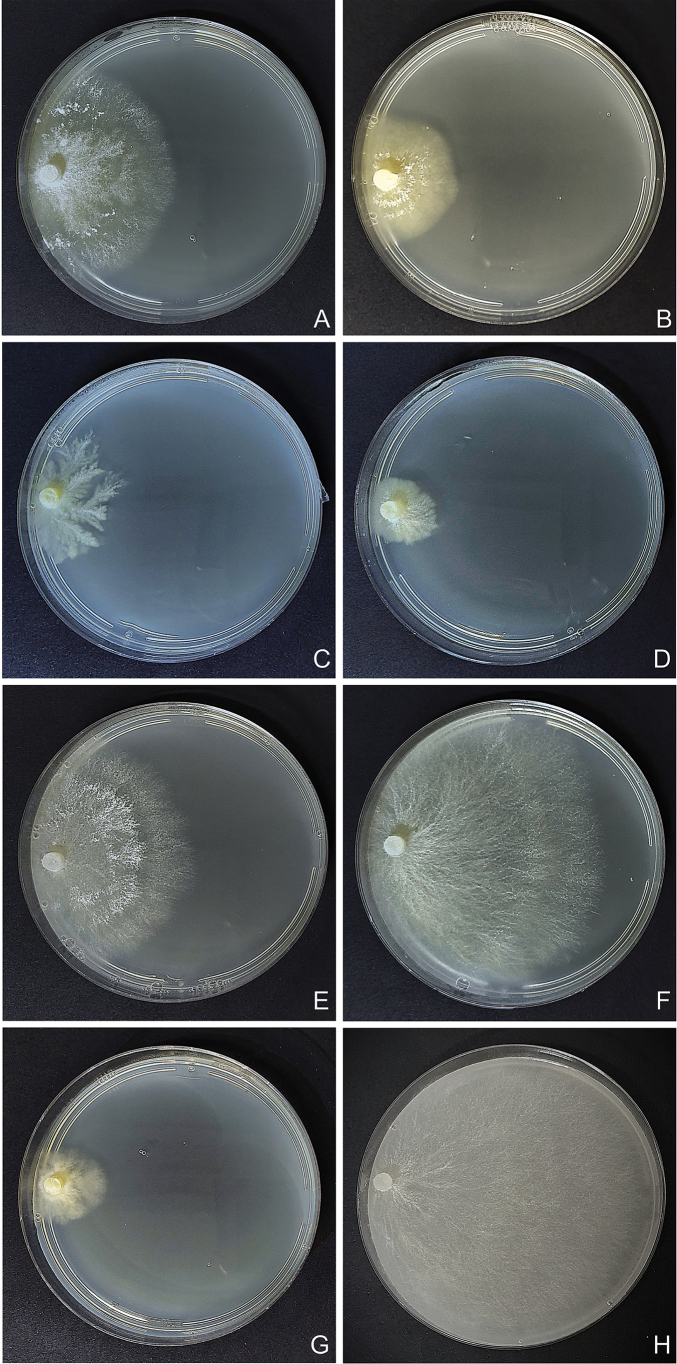
Macroscopic aspect of *Steccherinum* spp. cultures at six weeks. **A, B***S. bononiae*. **C, D***S. elegantissimum*. **E, F***S. molle*. **G, H***S. undulatum*. **A** 726/22. **B** NR71. **C** 720/21. **D** 721/21. **E** 734/22. **F** 739/23. **G** 743/23. **H** 760/24.

**Figure 6.**
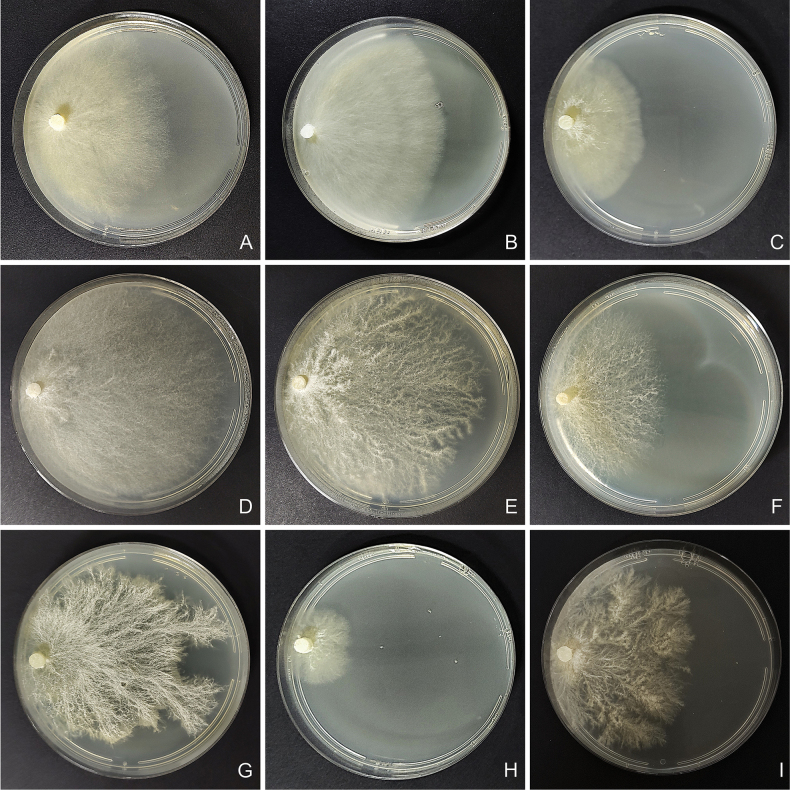
Macroscopic aspect of *Steccherinum* spp. cultures. **A–C***S. perparvulum*. **D–F***S. resinaceum*. **G–I***S. subochraceum*. **A** 710/20. **B** 742/23. **C** 744/23. **D** 551/17. **E** 665/19. **F** 679/19. **G** 730/22. **H** 748/23. **I** 761/24.

Regarding the culture studies carried out, all mycelia showed very similar micromorphology, with thin-walled, regularly clamped hyphae and abundant intercalary chlamydospores in the older areas of the mycelium, becoming scarcer in the advancing zone (Fig. 4). However, some specimens exhibited loss of clamp connections. Specimen NR71 presented only simple septa during the six-week study, in contrast to specimen 726/22, both belonging to *S. bononiae*, which displayed regularly clamped hyphae. Specimen 748/23 of *S. subochraceum* exhibited variation in the presence or absence of clamp connections, with a total loss of clamps after the third week of growth. However, the mycelium was checked again two months after the end of the study, and the hyphae were regularly clamped once more. Regarding the growth rates, specimens 742/23 (*S. perparvulum*), 730/22 (*S. subochraceum*), and 760/24 (*S. undulatum*) showed the highest rates, whereas specimens 721/21 (*S. elegantissimum*), 748/23 (*S. subochraceum*), and 743/23 (*S. undulatum*) showed the lowest rates (Table 3). Overall, all cultures presented a similar macromorphology, exhibiting thin mats with floccose mycelia (Figs 5, 6). In contrast, two specimens of *S. subochraceum* (730/22 and 761/24) presented strongly fimbriate and denser mycelia (Fig. 6G, I), similar to specimen 665/19 of *S. resinaceum* (Fig. 6E). Interestingly, *S. subochraceum* displayed notable intraspecific variation, with differences in both mycelial macromorphology and growth rates (Fig. 6G–I, Table 3).

**Table 3.** Average culture growth rates in studied *Steccherinum* spp.

		Week 1		Week 3		Week 6	
Species	Specimen	Avg. growth (mm)	Clamps	Avg. growth (mm)	Clamps	Avg. growth (mm)	Clamps
*Steccherinum bononiae*	NR71	8.2	-	35	-	73.5	-
	726/22	4.5	+	17	+	49.5	+
*S. elegantissimum*	720/21	1.7	+	19	+	39.2	+
	721/21	1.3	+	12	+	22.2	+
*S. molle*	734/22	10	+	40	+	68.2	+
	739/23	18.2	+	57	+	76	+
*S. perparvulum*	710/20	9	+	39	+	65	+
	742/23	13.2	+	52	+	79.3	+
	744/23	8	+	30	+	57.7	+
*S. resinaceum*	551/17	5.1	+	27.5	+	71.5	+
	665/19	9.5	+	31.5	+	65.7	+
	679/19	6.6	+	29	+	74	+
*S. subochraceum*	730/22	16	+	71	+	75.3	+
	748/23	4.5	+	16	+/-	30.5	-
	761/24	5.9	+	47	+	59	+
*S. undulatum*	743/23	0.8	+	13	+	36.25	+
	760/24	16	+	69.2	+	77.4	+

Full descriptions and comments on the new taxa are presented below, as well as discussions of species with newly obtained molecular data (*S. subochraceum* and *S. perparvulum*). A summary of the main features of morphologically similar hydnoid neotropical *Steccherinum* spp. is provided in Table 4, and the basidiospore measurements obtained in this study are shown in Table 5. An identification key to Brazilian corticioid species of *Steccherinum* and *Cabalodontia* is also presented. For poroid species of *Steccherinum*, see Westphalen et al. (2018).

**Table 4.** Main characteristics of studied odointioid/hydnoid Neotropical *Steccherinum* spp.

Species	Basidiome	Teeth	Spores (Lm x Wm)	Cystidia
*S. bononiae*	Resupinate to effused-reflexed. Corky to somewhat pliable. Pilei when present forming on the center and margins of the basidiomes.	Cylindrical, with acute to truncate apices, somewhat clustered and flattened. With an evident granulose aspect due to protuding cystidia; 4–6 per mm; Lm = 0.51 mm.	Ellipsoid. 3.1 × 2.0 µm.	Skeletocystidia elongated, covered with a thick cap of crystals, projecting above the hymenium; (20)35–65 × 5–9(10) µm.
*S. elegantissimum*	Resupinate. Forming small patches on the substratum that fuse as they grow. Membranaceous and pliable.	Conical to filiform, with acute apices, solitary, very thin and waxy; 6–8 per mm; Lm = 0.41 mm.	Ellipsoid. 4.2 × 2.5 µm.	Skeletocystidia elongated, covered with a cap of thin crystals, projecting above the hymenium; 20–70(75) x 4–8 µm.
*S. larssonii*	Resupinate to effused-reflexd. Pilei forming on the margins of the basidiomes. Waxy to corky.	Cylindrical, with round to acute apices, mostly solitary, 4–6 per mm; Lm = 0.5 mm.	Subglobose to broadly ellipsoid. 3.9 × 3.2 µm.	Skeletocystidia covered with a cap of crystals, protruding into the hymenium or slightly above it; 30–50(60) × 7–10 µm.
*S. molle*	Resupinate. Usually forming large patches. Soft to membranaceous, pliable and easily tearing.	Cylindrical to filiform, with acute apices., mostly solitary, 5–7 per mm. Lm = 0.45.	Ellipsoid. 3.1 × 2.1 µm.	Skeletocystidia coarsely encrusted with large crystals, immersed in the trama or with the apices protruding into the hymenium; (20)30–60 × 7–13(15) µm.
*S. perparvulum*	Resupinate to effused-reflexed. Papery and pliable, easily tearing. When present, small pilei forming on the margins of the basidiomes.	Conical to cylindrical, with acute apices, solitary; 4–7 per mm; Lm = 0.43 mm (reaching up to 0.7 mm in effused-reflexed basidiomes).	Subglobose to broadly ellipsoid. 2.7 × 1.9 µm.	Skeletocystidia covered with a cap of crystals, immersed in the trama or with the apices projecting above the hymenium; (20)25–42(48) × (4)6–12(15) µm.
*S. resinaceum*	Resupinate. Membranaceous to papery and pliable when flesh, turning waxy and tough upon drying.	Cylindrical, with truncate to acute apices, often bifurcate and laterally fused; clustered when dried. 5–6 per mm; Lm = 0.52.	Ellipsoid. 3.0 × 2.0 µm.	Skeletocystidia coarsely encrusted with large crystals, immersed in the trama or with the apices protruding into the hymenium; 25–45(50) × (7)9–11(12) µm.
*S. subochraceum*	Effused-reflexed, with imbricate pilei. Papery to corky and somewhat pliable.	Cylindrical, with truncate to acute apices, solitary; 3–5 per mm; Lm = 1.52.	Subglobose. 3.8 × 3.3 µm.	Skeletocystidia elongated, covered with a thin cap of crystals, immersed in the trama or more rarely projecting outwards; 25–50 × 5–8(9) µm.
*S. undulatum*	Effused-reflexed. With very small imbricate pilei forming a wavy pattern on the basidiomes.	Conical to cylindrical, with acute to round apices, sometimes flattened, solitary or arising from a common base; 4–6 per mm; Lm = 0.85 mm.	Ellipsoid. 3.6 × 2.3 µm.	Skeletocystidia covered with a cap of crystals, immersed in the trama or with the apices protruding into the hymenium; 20–60 × 7–10 µm.

**Table 5.** Basidiospore measurements of specimens studied.

Species/Specimen (voucher)	Length	Lm	Width	Wm	Q	Qm	n
*Cabalodontia albofulva* (563/17)	4.0–5.1(–5.3)	4.5	2.3–2.9(–3.1)	2.6	(1.60–)1.63–1.85(–1.88)	1.75	39
*Cabalodontia brunnea* (600/17)	(3.8–)3.9–4.9	4.3	2.4–3.0(–3.1)	2.8	(1.38–)1.39–1.76(–1.78)	1.56	36
*Steccherinum bononiae*	2.6–3.6	3.1	1.8–2.5	2	1.38–1.65	1.51	137
NR71	(2.8–)3.0–3.5	3.2	(1.8–)1.9–2.3(–2.4)	2.1	1.43–1.60(–1.63)	1.54	24
547/17	(2.5–)2.8–3.4(–3.5)	3	1.8–2.4	2	(1.39–)1.40–1.63	1.51	25
557/17	(2.7–)2.8–3.6	3.1	1.9–2.3(–2.4)	2	(1.38–)1.40–1.63(–1.63)	1.52	34
AL1615	(2.8–)2.9–3.3	3.1	1.9–2.2	2.1	(1.43–)1.45–1.58(–1.60)	1.5	30
726/22	(2.7–)2.8–3.2(–3.4)	3	(1.8–)1.9–2.3(–2.4)	2.1	1.38–1.60	1.46	24
*S. elegantissimum*	(3.4–)3.5–5.0(–5.1)	4.2	2.2–2.9(–3.0)	2.5	(1.42–)1.35–1.88(–1.92)	1.64	94
633/18	(3.4–)3.5–4.0	3.7	2.2–2.5(–2.7)	2.4	1.48–1.68(–1.73)	1.57	20
720/21	(3.5–)3.6–5.0(–5.1)	4.4	(2.2–)2.3–2.9(–3.0)	2.6	1.42–)1.43–1.88(–1.92)	1.66	50
721/21	(3.8–)3.9–4.6(–4.7)	4.2	(2.3–)2.4–2.6(–2.7)	2.5	(1.50–)1.52–1.81	1.68	24
*S. larssonii*	3.5–4.3(–4.5)	3.9	3–3.7(–4.0)	3.2	(1.13–)1.15–1.28(–1.30)	1.21	220
KHL11326	3.3–3.9(–4.0)	3.6	2.7–3.3	3	1.16–1.24(–1.26)	1.2	15
KHL11622	(3.5–)3.6–4.4(–4.6)	3.9	3.0–3.6(–3.9)	3.2	(1.10–)1.13–1.28	1.21	20
KHL9806	(3.6–)3.7–4.2(–4.3)	3.9	(3.0–)3.1–3.7(–3.9)	3.4	(1.10–)1.15–1.22(–1.23)	1.16	15
LR23000	(3.6–)3.7–3.9	3.8	2.8–3.1(–3.3)	3	(1.15–)1.19–1.33(–1.36)	1.27	15
LR23024	(3.6–)3.7–4.2	3.9	(3.1–)3.2–3.6	3.4	(1.10–)1.12–1.22(–1.24)	1.16	15
MV634	(3.6–)3.7–4.3(–4.5)	3.9	2.9–3.4(–3.50)	3.1	(1.17–)1.19–1.35(–1.38)	1.28	35
MWC593/17	(3.5–)3.7–4.4(–4.5)	4	3.0–3.6(–3.80)	3.3	(1.14–)1.17–1.27	1.21	25
MWC594/17	3.6–4.3(–4.5)	3.9	(2.7–)2.0–3.5(–3.6)	3.2	1.14–1.34(–1.40)	1.23	25
MWC621/17	(3.5–)3.6–4.2(–4.3)	3.9	3.1–3.6(–3.8)	3.4	(1.05–)1.10–1.22	1.15	30
MWC676/19	3.5–4.4(–4.6)	3.9	(2.7–)2.8–3.4(–3.9)	3.1	(1.16–)1.18–1.34(–1.37)	1.25	25
*S. molle*	2.5–3.5(–3.6)	3.1	1.8–2.4(–2.5)	2.1	1.20–1.60	1.45	113
641/18	3.0–3.5(–3.6)	3.3	2.0–2.4	2.3	(1.41–)1.42–1.57(–1.60)	1.47	32
661/18	3.1–3.5(–3.6)	3.3	2.0–2.4	2.2	(1.43–)1.45–1.60	1.49	19
734/22	2.9–3.3	3.1	2.0–2.3	2.1	1.36–1.55(–1.58)	1.46	25
739/23	(2.9–)3.0–3.6	3.3	(2.0–)2.1–2.4(–2.5)	2.3	(1.33–)1.36–1.52(–1.58)	1.46	11
MV450	(2.4–)2.5–2.9(–3.0)	2.7	1.8–2.0(–2.1)	1.9	(1.20–)1.25–1.53(–1.56)	1.38	26
*S. perparvulum*	2.3–3.0(3.2)	2.7	1.7–2.2	1.9	1.25–1.55(1.58)	1.42	204
524/17	2.3–2.9(–3.0)	2.6	1.7–2.2	1.9	1.25–1.42	1.34	30
LR24589 (holotype)	2.3–2.9(–3.0)	2.6	1.7–2.1(–2.2)	1.9	(1.29–)1.30–1.47(–1.50)	1.38	35
659/18	2.6–3.0(–3.1)	2.8	(1.7)1.8–2.1	1.9	(1.38–)1.40–1.53	1.46	35
MV815	2.3–2.8(–2.9)	2.6	1.7–2.1(–2.2)	1.9	(1.27–)1.30–1.50(–1.53)	1.4	30
592/17	2.7–3.2	3	(1.8–)1.9–2.2	2	(1.30–)1.33–1.53	1.46	34
710/20	(2.5–)2.6–3.0	2.8	1.8–2.1	1.9	1.37–1.53(1.58)	1.44	25
742/23	(2.6–)2.7–2.9	2.8	1.9–2.1	2	(1.37–)1.38–1.47(–1.53)	1.42	15
*S. resinaceum*	2.7–3.4(–3.5)	3	1.7–2.4	2	1.38–1.59(–1.60)	1.49	52
551/17	2.8–3.4(–3.5)	3.1	1.8–2.4	2.1	1.38–1.58(–1.60)	1.47	35
665/19	2.7–3.0	2.8	1.7–2.0(–2.1)	1.8	(1.38–)1.42–1.59	1.54	17
*S. subochraceum*	(3.2–)3.3–4.2	3.8	2.5–3.8	3.3	1.06–1.24(–1.30)	1.15	93
PACA 22824	3.7–4.0	3.8	3.0–3.5	3.2	1.14–1.23	1.19	5
SP 97591 (holotype)	(3.2–)3.4–3.8(–4.0)	3.6	2.5–3.5	3.1	(1.06–)1.09–1.23(–1.30)	1.15	20
730/22	(3.3–)3.4–4.1	3.8	(2.9–)3.0–3–8(–4.1)	3.4	(1.06–)1.08–1.19(–1.20)	1.13	32
746/23	(3.5–)3.7–4.2	3.9	(3.0–)3.2–3.7	3.4	1.11–1.24(–1.29)	1.16	24
748/23	3.2–3.9	3.6	2.6–3.4	3.1	(1.12–)1.14–1.19(–1.22)	1.16	12
*S. undulatum*	3.3–4.1	3.6	2.0–2.7(–2.8)	2.3	(1.37–)1.39–1.65(–1.67)	1.52	72
743/23	3.3–3.8	3.5	2.0–2.6	2.3	1.42–1.67	1.54	38
760/24	(3.2–)3.3–3.8(–4.7)	3.5	2.1–2.7(–2.8)	2.4	(1.37–)1.39–1.57(–1.64)	1.5	34

### Taxonomy

#### Cabalodontia albofulva

Taxon classificationAnimaliaPolyporalesSteccherinaceae

Westphalen & Gugliotta sp. nov.

8A3C1C10-5A46-5595-8925-2C6A8D0C996E

861265

**Figure 7.**
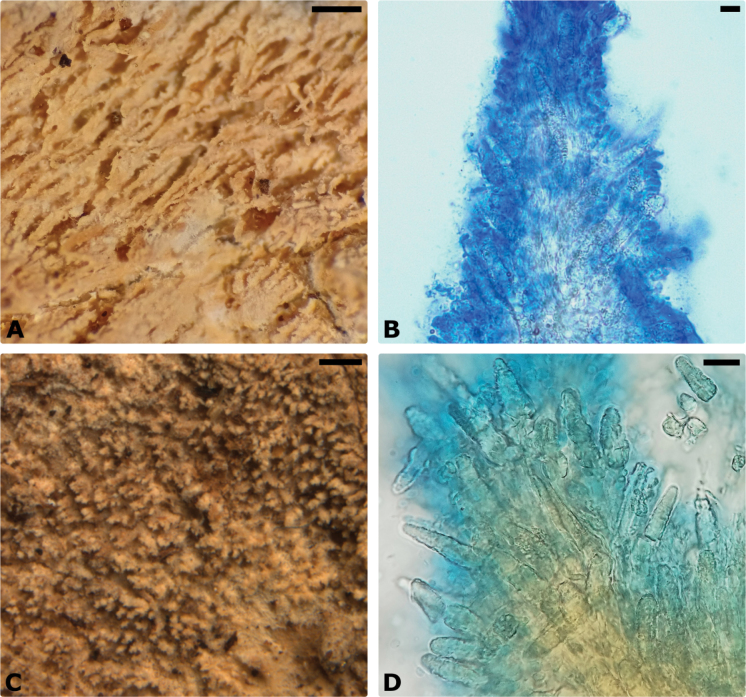
Macroscopic and microscopic aspects of *Cabalodontia* spp. **A, B***C. albofulva* (563/17). **C, D***C. brunnea* (600/17). Scale bars: 0.5 mm (**A, C**); 10 µm (**B, D**).

Figs 7A, 7B, 11A, 11J

##### Etymology.

Refers to the white basidiomes that become yellowish when dried.

**Figure 8.**
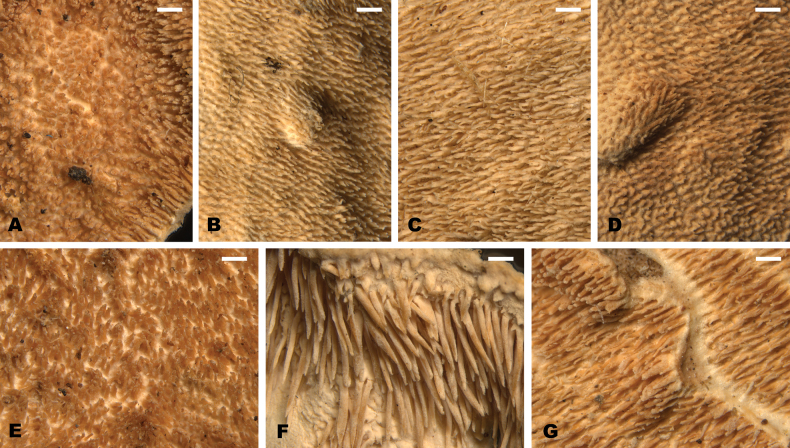
Aculei in hymenophores of Neotropical *Steccherinum* spp. **A***S. bononiae* (726/22). **B***S. elegantissimum* (721/21). **C***S. molle* (734/22). **D***S. perparvulum* (710/20). **E***S. resinaceum* (665/19). **F***S. subochraceum* (746/23). **G***S. undulatum* (743/23). Scale bars: 0.5 mm.

**Figure 9.**
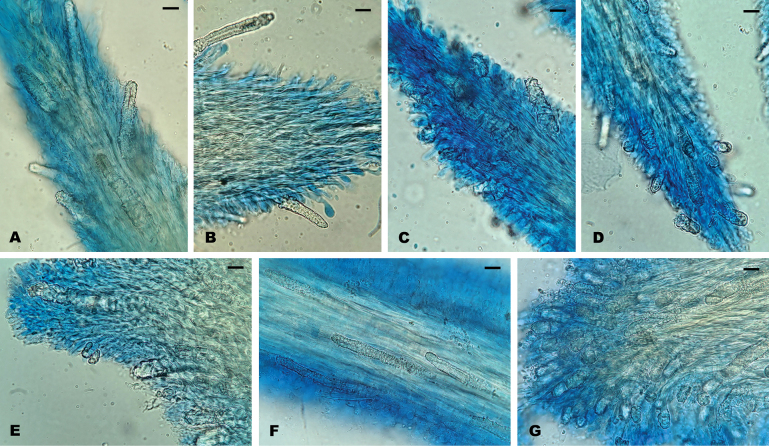
Detail of the trama and cystidia of Neotropical *Steccherinum* spp. **A***S. bononiae* (726/22). **B***S. elegantissimum* (721/21). **C***S. molle* (734/22). **D***S. perparvulum* (710/20). **E***S. resinaceum* (665/19). **F***S. subochraceum* (746/23). **G***S. undulatum* (743/23). Scale bars: 10 µm.

**Figure 10.**
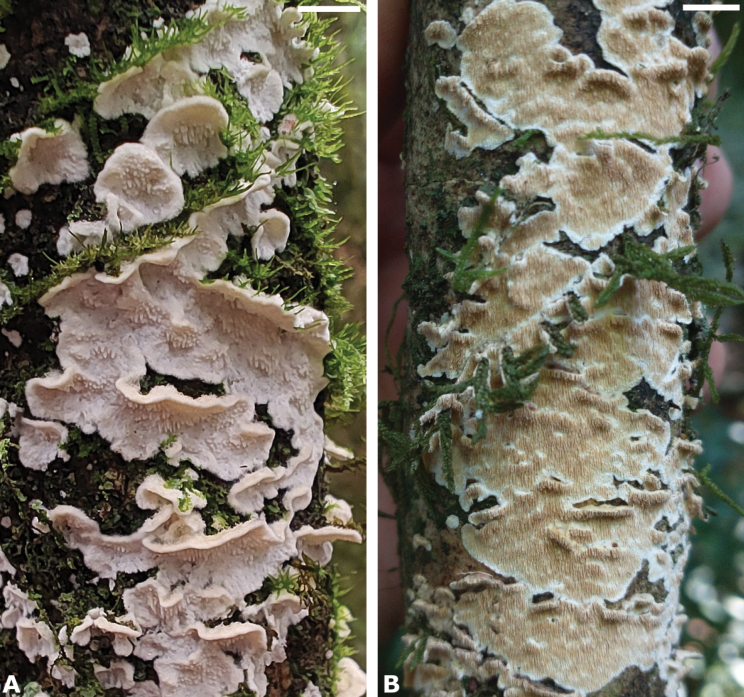
Fresh basidiomes in situ. **A***S. subochraceum*. **B***S. undulatum*. Scale bars: 0.5 cm.

**Figure 11.**
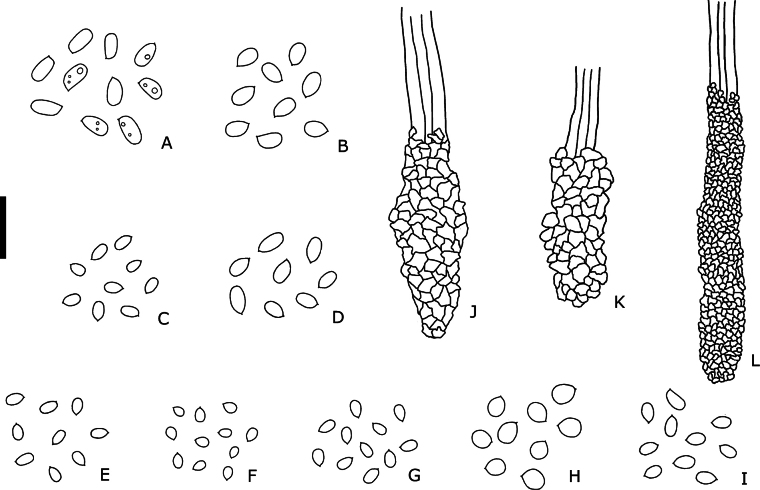
Microscopic features of Neotropical *Steccherinum* and *Cabalodontia* species. **A–I** Basidiospores. **J–L** Skeletocystidia. **A***C. albofulva*. **B***C. brunnea*. **C***S. bononiae*. **D***S. elegantissimum*. **E***S. molle*. **F***S. perparvulum*. **G***S. resinaceum*. **H***S. subochraceum*. **I***S. undulatum*. **J** Coarsely encrusted tapering cystidia (*C. brunnea*, 600/17). **K** Skeletocystidia encrusted with large crystals (*S. molle*, 734/22). **L** Skeletocystidia covered with thin crystals (*S. elegantissimum*, 721/21). Scale bar: 10 µm.

##### Diagnosis.

Differs from *C. queletii* by thinner and more fragile aculei and smaller basidiospores.

##### Typification.

Brazil. Rio Grande do Sul: São Francisco de Paula, Parador Hampel, 19 Jun. 2017, M.C. Westphalen 563/17 (SP 467080).

##### Description.

Basidiomes adnante, annual, resupinate, not detaching, forming small patches on the substrate, very soft and brittle; sterile margins white, membranaceous, up to 1 mm wide. Hymenophore odontioid, white to cream when fresh, becoming yellowish to beige upon drying. Aculei thin, up to 0.8 mm long, somewhat clustered and very brittle upon drying, 6–8 per mm. Subiculum, white to cream, homogeneous, cottony, thin and fragile, up to 0.5 mm thick.

Hyphal system monomitic, hyphae loosely arranged; generative hyphae clamped, thin- to slightly thick-walled, hyaline, often branching near the septa, 3–5 μm wide, CB–. Skeletocystidia abundant, arising from sclerified generative hyphae in the subiculum and projecting into the trama and above the hymenium, clavate, usually tapering towards the apex, encrusted portion 25–60(–70) × 6–11 μm; thin-walled leptocystidia present on tips of the aculei, clavate to capitate, 4–7 μm wide. Basidia clavate, tetrasterigmate, 12–15 × 3.5–5 μm. Basidiospores ellipsoid, hyaline, thin-walled, IKI–, CB–, (4–)4.3–5.1(–5.3) × 2.4–3.0(–3.1) μm.

##### Habitat and distribution.

Known only from the type locality in Araucaria forests in Rio Grande do Sul State. Growing on dead logs of unidentified angiosperms.

##### Notes.

*Cabalodontia albofulva* is characterized by fragile basidiomes with thin aculei measuring 0.5–0.8 mm long and ellipsoid basidiospores measuring (4–)4.3–5.1(–5.3) × 2.4–3.0(–3.1) µm. *Cabalodontia queletii* (Bourdot & Galzin) Piątek is morphologically similar but differs by having thicker aculei and larger basidiospores, measuring 5–6 × 3–3.5 µm (Bernicchia and Gorjón 2010). Phylogenetically, *C. albofulva* is closely related to *C. lincangense*, from which it differs by approximately eight bp in the ITS sequences, four in the *tef*1-α coding region, and five in the *rpb*1 coding region. Morphologically, however, *C. lincangense* is distinct, displaying more widely spaced and longer aculei (2–4 per mm, 1–1.5 mm long) and shorter basidiospores measuring (3.5–)3.8–4.2(–4.5) × (2.3–)2.5–2.8 µm (Dong et al. 2024). *Cabalodontia albofulva* is currently known only from its type locality in southern Brazil. Despite extensive sampling in the surrounding region, including adjacent Araucaria forests, no additional specimens have been collected, suggesting that it could represent a rare taxon.

#### Cabalodontia brunnea

Taxon classificationAnimaliaPolyporalesSteccherinaceae

Westphalen & Regio sp. nov.

D92BD90A-E142-5A88-AFB4-6AB82AD3C90D

861267

Figs 7C, 7D, 11B

##### Etymology.

Refers to the brownish basidiomes.

##### Diagnosis.

Characterized by the pale brown basidiomes with short, irregular spines, hyaline to yellow hyphae, and basidiospores (3.7–)4–4.7(–4.9) × 2.4–3.0(–3.1) μm.

##### Typification.

Brazil. Rio Grande do Sul: Caxias do Sul, Cânion Palanquinhos, 18 Sep. 2017, M.C. Westphalen 600/17 (SP 512588).

##### Description.

Basidiomes adnate, resupinate, annual, not detaching, forming confluent large patches on the substrate, soft and somewhat brittle; sterile margins absent or very thin, fimbriate to byssoid, up to 1 mm wide. Hymenophore pale brown to ochraceous when fresh, unchanging or slightly darker upon drying, formed by small irregular spines, up to 0.5 mm long, often somewhat cluttered, very brittle upon drying, 6–9 per mm. Subiculum beige to pale yellowish brown, homogeneous, thin and fragile, cottony, up to 0.5 mm thick.

Hyphal system monomitic, hyphae more densely arranged towards the trama and looser near the substratum; generative hyphae clamped, thin- to thick-walled, often branching near the septa or at clamp connections, 2.5–5 μm wide, hyaline to pale yellow, golden yellowish-brown in mass, CB+. Skeletocystidia abundant, heavily encrusted, arising from sclerified generative hyphae in the subiculum and projecting toward the trama and above the hymenium, clavate or more commonly tapering towards the apex, encrusted portion 25–70(–100) × 5–9(–12) μm. Basidia clavate, tetrasterigmate, 13–16 × 4–5 μm. Basidiospores ellipsoid, hyaline, thin-walled, (3.7–)4–4.7(–4.9) × 2.4–3.0(–3.1) μm, IKI–, CB–.

##### Habitat and distribution.

Known only from the type locality in Araucaria forests in Rio Grande do Sul State. Growing on dead logs of unidentified angiosperms.

##### Notes.

*Cabalodontia brunnea* is distinguished within the genus by its pale brown basidiomes with irregular short spines and a somewhat farinaceous appearance due to the encrusted cystidia. Microscopically, it presents yellowish hyphae and ellipsoid basidiospores measuring (3.8–)4–4.7(–4.9) × 2.4–3.0(–3.1) μm. As in the case of *C. albofulva*, this species is known only from its type locality in southern Brazil. Despite extensive sampling in the region, including nearby Araucaria forests, no additional specimens have been found, suggesting that it could represent a rare taxon.

Phylogenetically, *C. brunnea* is closely related to *C. delicata*, a species also found in Araucaria forests and high-altitude Atlantic rainforest areas (Westphalen et al. 2019). However, *C. delicata* is relatively common in these habitats and differs from *C. brunnea* by its pale white to cream-colored basidiomes, shorter aculei, and smaller, subglobose to broadly ellipsoid basidiospores.

#### Cabalodontia lincangense

Taxon classificationAnimaliaPolyporalesSteccherinaceae

(J.H. Dong & C.L. Zhao) Westphalen & Regio comb. nov.

468D4A42-6770-5C55-95F4-07B47008A6D5

861268

##### Basionym.

*Steccherinum lincangense* J.H. Dong & C.L. Zhao, Mycosphere 15 (1): 1252. 2024.

##### Notes.

*Cabalodontia lincangense* was originally described based on a phylogeny that did not adequately represent *Cabalodontia* and other *Steccherinaceae* lineages, resulting in its placement in *Steccherinum*. In our analysis, the species nests within *Cabalodontia*, sharing morphological features such as an odontioid hymenophore, a monomitic hyphal system with clamped hyphae, and skeletocystidia tapering toward the apex. It is closely related to *C. albofulva* but can be differentiated by more widely spaced and longer aculei (2–4 per mm, 1–1.5 mm long) and shorter basidiospores measuring (3.5–)3.8–4.2(–4.5) × (2.3–)2.5–2.8 µm (Dong et al. 2024).

#### Cabalodontia tenuissima

Taxon classificationAnimaliaPolyporalesSteccherinaceae

(C.L. Zhao & Y.X. Wu) Westphalen & Regio comb. nov.

ABB50256-A31B-533B-B19C-5DB64668F5C7

861269

##### Basionym.

*Steccherinum tenuissimum* C.L. Zhao & Y.X. Wu, PLoS ONE 16 (1): e0244520, 7. 2021.

##### Notes.

*Cabalodontia tenuissima* was originally described based on a phylogeny that included only *Steccherinum* spp., where it nested in a separate clade with *S. xanthum*, a species shown by our analyses to be a synonym of *C. albofibrillosa*. Morphologically, *C. tenuissima* differs from all other confirmed *Cabalodontia* spp. by presenting a dimitic hyphal system, although it shares other features of the genus, such as fragile and thin basidiomes with an odontioid hymenophore (Wu et al. 2021a). It is possible that the skeletal hyphae reported by the authors represent undifferentiated lower portions of skeletocystidia, since the incrustations are restricted to the apical region. This interpretation is also supported by the drawings presented in the original description, where clamped hyphae are abundant and few thick-walled skeletal hyphae are shown, which is not common in truly dimitic species. Such thick-walled hyphal segments are also observed in other species of the genus, such as *C. brunnea* and *C. albofulva*, but they are scarce and, when carefully observed, give rise to cystidia. Further examination of the specimens is required to confirm this hypothesis. Nevertheless, because our phylogenetic analyses are well supported and include sequences from the type material (CLZhao 3153), we chose to transfer this species to *Cabalodontia*. Together with *Irpex oreophilus* (Lindsay & Gilb.) Niemelä, these are the only two dimitic taxa recovered within the genus.

#### Steccherinum bononiae

Taxon classificationAnimaliaPolyporalesSteccherinaceae

Westphalen & Gugliotta sp. nov.

17B1C77B-D1D8-52CE-A66B-FD9B1EF5B93C

858851

Figs 8A, 9A, 11C

##### Etymology.

In honor of Dr. Vera Bononi, for her contribution to the knowledge of *Steccherinum* in Brazil.

##### Diagnosis.

Distinguished by the combination of resupinate to effused-reflexed basidiomes with waxy spines upon drying, elongated skeletocystidia protruding above the hymenium, aculei measuring up to 0.75 mm, and basidiospores 2.6–3.6 × 1.8–2.5 µm.

##### Typification.

Brazil. São Paulo: São Luis do Paraitinga, Parque Estadual da Serra do Mar, Núcleo Santa Virgínia, Trilha Poço do Pito, 06 Jun. 2017, M.C. Westphalen 557/17 (SP 512686).

##### Description.

Basidiomes adnate, resupinate to effused-reflexed, easily separable from the substratum and usually detaching upon drying, membranaceous to papery and pliable when fresh, turning corky and somewhat waxy upon drying; pilei when present arising from effused parts of the basidiomes and at the margins, usually imbricate and narrow, up to 8 mm wide, pileus surface cream to beige, fimbriate, faintly zonate; sterile margins entire, smooth, pelliculose, up to 2 mm wide, white to cream. Hymenophore hydnoid, pale salmon to pale yellowish when fresh, turning beige to tan upon drying, aculei 0.25–0.75 × 0.1–0.25 mm, with acute apices, with a pilose appearance from the protruding cystidia, solitary or more rarely laterally fused, somewhat crowded, 6–9 per mm. Subiculum cream to beige, homogeneous, slightly dense, up to 0.8 mm thick.

Hyphal system dimitic; subicular hyphae compact, not agglutinated; aculei tramal hyphae intertwined, parallel; generative hyphae clamped, thin to slightly thick-walled, hyaline, occasionally branched, 2–4 µm wide, more abundant in the base of the subiculum; skeletal hyphae thick-walled to almost solid, hyaline to slightly yellowish, 2–4.5 µm wide. Skeletocystidia abundant, arising from the trama and protruding above the hymenium, somewhat elongated, covered with medium-sized crystals, (20–35–65 × 5–9(–10) µm; leptocystidia present, often scattered and more abundant at the apical portion of the aculei, clavate to fusoid, smooth to apically encrusted. Basidia clavate, tetrasterigmate, 10–14 × 4–5 µm. Basidiospores broadly ellipsoid, hyaline, thin-walled IKI–, CB–, 2.6–3.6 × 1.8–2.5 µm.

##### Mating system.

Tetrapolar. Monosporic cultures obtained from specimen NR71.

##### Habitat and distribution.

Known from southern, southeastern, and northeastern Brazil. Likely widespread in the Brazilian Atlantic Rainforest.

##### Specimens examined.

Brazil • Pernambuco: Olinda, 7 GAC - Batalhão do Exército, 16 Jun. 2018, R.S. Chikowski RC1625 (URM 93107). • Rio Grande do Sul: São Francisco de Paula, FLONA, 12 Mar. 2022, M.C. Westphalen 726/22 (ICN 213868); • Dom Pedro de Alcântara, RPPN Mata do Prof. Baptista, 17 Nov. 2022, N.C. Regio NR71 (ICN 213869). • São Paulo: São Paulo, Parque Estadual das Fontes do Ipiranga, 09 May 2014, A.M. Gugliotta 1615 (SP 512683); • Parque Estadual Cantareira, Núcleo Engordador, Trilha da Cachoeira, 24 Apr. 2017, M.C. Westphalen 547/17 (SP 512685); • Ribeirão Grande. Parque Estadual Intervales, Trilha roda d’água, 07 Jul. 2015, V. Motato-Vásquez MV446 (SP 512675).

##### Notes.

Basidiomes of *S. bononiae* exhibit considerable variation, ranging from completely effused to effused-reflexed, and aculei vary in size from 0.25 to 0.75 mm long. The basidiospore size and shape in this species resemble those of *S. molle* and *S. resinaceum*. However, *S. molle* can be distinguished by its softer, membranaceous basidiomes, slightly shorter spines, and wider skeletocystidia, which are more deeply embedded in the trama and covered with larger, chunky crystals. *Steccherinum resinaceum*, in turn, can be distinguished by its denser basidiomes, laterally fused aculei that are often bifurcated at the apices, and embedded cystidia covered with large crystals. Phylogenetically, *S. bononiae* forms a sister clade to *S. elegantissimum*, but the latter can be distinguished by its thinner, shorter aculei and larger basidiospores. Additionally, *S. elegantissimum* typically grows on thin branches, forming small concrescent patches, whereas *S. bononiae* usually forms basidiomes as a single patch. Both species share the presence of elongated and projecting cystidia covered with a somewhat organized cap of crystals, but these crystals are slightly larger in *S. bononiae* (Fig. 9).

#### Steccherinum elegantissimum

Taxon classificationAnimaliaPolyporalesSteccherinaceae

Westphalen & R.M. Silveira sp. nov.

EA3F572F-5228-56E3-B8A2-7CAA67DB4127

858852

Figs 8B, 9B, 11D, 11L

##### Etymology.

Refers to the delicate basidiomes with thin aculei and the elongated projecting cystidia.

##### Diagnosis.

Characterized by effused basidiome with small aculei (up to 0.5 mm long), a cottony subiculum, elongated and thin skeletocystidia protruding above the hymenium, and basidiospores with (3.4–)3.5–5.0(–5.1) × 2.2–2.9(–3.0) µm.

##### Typification.

Brazil. São Paulo: Ribeirão Grande. Parque Estadual Intervales, 28 Feb. 2018, M.C. Westphalen 633/18 (SP 512681).

##### Description.

Basidiomes adnate, effused, usually not detaching when dried, but easily separable if pulled from the substratum, formed by the coalescence of several small patches, membranaceous and pliable when fresh, turning papery and fragile after drying; sterile margins entire, byssoid, smooth to finely fimbriate, thin, up to 0.5 mm wide. Hymenophore hydnoid, cream to pale salmon when fresh, more or less unchanging or slightly duller upon drying, aculei 0.3–0.5 (0.55) × 0.1–0.2 mm, with acute apices, pilose from the protruding cystidia, solitary or rarely fused at the base, sub-distant, 5–8 per mm. Subiculum white, homogeneous, with a loose cottony and soft consistency, up to 0.3 mm thick.

Hyphal system dimitic, subicular hyphae loosely arranged, tramal hyphae intertwined and somewhat more densely arranged; generative hyphae clamped, thin to slightly thick-walled, hyaline, occasionally branched, 2–3 µm wide; skeletal hyphae thick-walled to almost solid, abundant throughout the basidiome, hyaline, 2–4 µm wide. Skeletocystidia abundant, arising from the trama and protruding above the hymenium, somewhat thin and elongated, covered with small crystals, 20–70(–75) × 4–8, but rarely above 6 µm wide; leptocystidia present, often scattered and more abundant at the apical portion of the aculei, clavate to fusoid, smooth. Basidia clavate, tetrasterigmate, 13–15 × 4.5–5 µm. Basidiospores ellipsoid to narrowly ellipsoid, hyaline, thin-walled, IKI–, CB–, (3.4–)3.5–5.0(–5.1) × 2.2–2.9(–3.0) µm.

##### Mating system.

Tetrapolar. Monosporic cultures obtained from specimens 720/21 and 721/21.

##### Habitat and distribution.

Known from Araucaria forest and Atlantic Rainforest areas in southern and southeastern Brazil.

##### Specimens examined.

Brazil • Rio Grande do Sul: Canela, FLONA, 22 Oct. 2021, M.C. Westphalen 720/21 and 721/21 (ICN 213870 and 213871).

##### Notes.

This species can be recognized by its small, sub-distant aculei and basidiomes formed by several concrescent small patches, typically growing on thin branches. Microscopically, the basidiospores are larger compared to other Neotropical *Steccherinum* species, measuring (3.4–)3.5–5.0(–5.1) × 2.2–2.9(–3.0) µm. Additionally, *S. elegantissimum* has the longest and thinnest cystidia in the group, covered by a layer of thin crystals (Figs 9B, 11L). The basidiomes with a cottony subiculum resemble those of *S. perparvulum* and *S. molle*, but the latter two species have smaller basidiospores and typically form larger patches that cover wider branches or logs.

#### Steccherinum molle

Taxon classificationAnimaliaPolyporalesSteccherinaceae

Westphalen & Minosso sp. nov.

374EBD76-0E29-51DE-AD9A-CBEE1650DB00

858854

Figs 8C, 9C, 11E, 11K

##### Etymology.

Refers to the soft and pliable consistency of the basidiomes.

##### Diagnosis.

Recognized by soft basidomes with a cottony subiculum, small aculei up to 0.7 mm long, skeletocystidia usually embedded in the trama and encrusted with large crystals, and basidiospores 2.5–3.5(–3.6) × 1.8–2.4(–2.5) µm.

##### Typification.

Brazil. Rio Grande do Sul: Dom Pedro de Alcântara, RPPN Mata do Prof. Baptista, 10. Sep. 2022, M.C. Westphalen 734/22 (ICN 213874).

##### Description.

Basidiomes adnate, resupinate, easily separable from the substratum, usually detaching upon drying, coalescing to form large patches, soft and membranaceous when fresh, unchanging to slightly papery upon drying, but remaining soft and pliable, easily tearing; sterile margins thin, cottony, smooth to finely floccose, up to 1.5 mm wide. Hymenophore hydnoid, cream to pale orange when fresh, unchanged upon drying, aculei (0.3–)0.4–0.5(–0.7) × 0.1–0.2(–0.25) mm, with acute apices, sometimes slightly asperulate from the protuding cystidia, solitary or more rarely fused at the base, sub-distant, 5–7 per mm. Subiculum white, homogeneous, loose, and cottony, very thin, up to 0.4 mm thick.

Hyphal system dimitic, subicular hyphae very loosely arranged, tramal hyphae intertwined and somewhat densely arranged, subparallel; generative hyphae clamped, thin to slightly thick-walled, hyaline, occasionally branched, 2–3 µm wide; skeletal hyphae thick-walled to almost solid, hyaline, 2–4 µm wide. Skeletocystidia abundant, immersed in the trama or protruding into the hymenium, coarsely encrusted with large crystals, (20–)30–60 × 7–13(–15); leptocystidia present, more commonly seen on the apices of the aculei, smooth to finely encrusted, mostly clavate. Basidia clavate, tetrasterigmate, 10–14 × 4–5 µm. Basidiospores ellipsoid, hyaline, thin-walled, IKI–, CB–, 2.5–3.5(–3.6) × 1.8–2.4(–2.5) µm.

##### Mating system.

Tetrapolar. Monosporic cultures obtained from specimen 734/22.

##### Habitat and distribution.

Known from Atlantic Rainforest areas in southeastern and northeastern Brazil and Araucaria Forests in southern Brazil. Mostly found in high-altitude regions above 700 m. Likely widespread throughout the Atlantic rainforest biome.

##### Specimens examined.

Brazil • Rio Grande do Sul: São Francisco de Paula, Hotel Parador Hampel, Trilha, 19 Jun. 2017, M.C. Westphalen 568/17 (SP 512692); • ibid., FLONA, 14 May 2018, M.C. Westphalen 641/18 (SP512689); • Farroupilha, Parque dos Pinheiros, 20 Apr. 2018, M.C. Westphalen 661/18 (SP 512690); • ibid., 27 Mar. 2019 M.C. Westphalen 687/19 (SP 512691); • ibid., 21 Jan. 2023 M.C. Westphalen 739/23 (ICN 213875). • São Paulo: Ribeirão Grande, Parque Estadual Intervales, Trilha da gruta, 07 Jul. 2015, V. Motato-Vásquez MV450 (SP 512672). • Sergipe: Areia Branca, Parque Nacional Serra de Itabaiana, 25 Jul. 2025, R.S. Souza RSS236 (URM).

##### Notes.

This species can be recognized mainly by the soft and pliable basidiomes with a cottony subiculum, usually forming large patches on the substratum, small aculei, and embedded cystidia encrusted with large crystals. *Steccherinum resinaceum* presents similar basidiospores and cystidia but can be distinguished by the harder, waxy basidiomes and laterally fused or bifurcate aculei. *Steccherinum perparvulum* is similar macroscopically but presents papery basidiomes upon drying, not soft or pliable, shorter basidiospores, and slightly thinner cystidia encrusted with smaller crystals.

#### Steccherinum perparvulum

Taxon classificationAnimaliaPolyporalesSteccherinaceae

Hjortstam & Ryvarden

0A01D71B-AC67-55D2-8EB9-CF34490005C2

Figs 8D, 9D, 11F

##### Description.

Full description in: Hjortstam and Ryvarden (2008).

##### Mating system.

Tetrapolar. Monosporic cultures obtained from specimens 710/20 and 742/23.

##### Habitat and distribution.

Widespread in southern to southeastern Brazil, mainly in high-altitude areas.

##### Specimens examined.

Brazil • Rio Grande do Sul: Nova Roma do Sul, ponte velha, 05 Apr. 2017, M.C. Westphalen 524/17 (SP512658); • São Francisco de Paula, CPCN Pró-Mata, 17 May 2018, M.C. Westphalen 659/18 (SP 512661); • ibid., 22. Apr. 2023 M.C. Westphalen 744/23 (ICN 213878); • ibid., 19 Apr; 2024, N.C. Regio NR186 (ICN 213879); • ibid., FLONA, 19 Apr. 2023, M.C. Westphalen 742/23 (ICN 213877); • Caxias do Sul, Parque da Gruta Nossa Senhora de Lourdes, 28 Mar. 2019, M.C. Westphalen 692/19; ibid., 09. Nov. 2020, M.C. Westphalen 710/20 (ICN 213876). • São Paulo: Moji-Guaçu, Fazenda Campininha, 29–30 Jan 1987, D. Pegler, K. Hjortstam & L. Ryvarden 24589 (O - holotype); • Parelheiros, Parque Estadual da Serra do Mar, Núcleo Curucutu, 16 Nov. 2016, V. Motato-Vásquez MV815 (SP 512656); • São Paulo, Parque CIENTEC, 07 Jul. 2016, V. Motato-Vásquez MV728 (SP 512657); • ibid., Parque Estadual Cantareira, Núcleo Engordador, Trilha da Cachoeira, 24 Apr. 2017, M.C. Westphalen 543/17 (SP 512659); • Santo André, Reserva Biológica do Alto da Serra de Paranapiacaba, 25 Aug. 2017, M.C. Westphalen 592/17 (SP 512660).

##### Notes.

**Figure 12.**
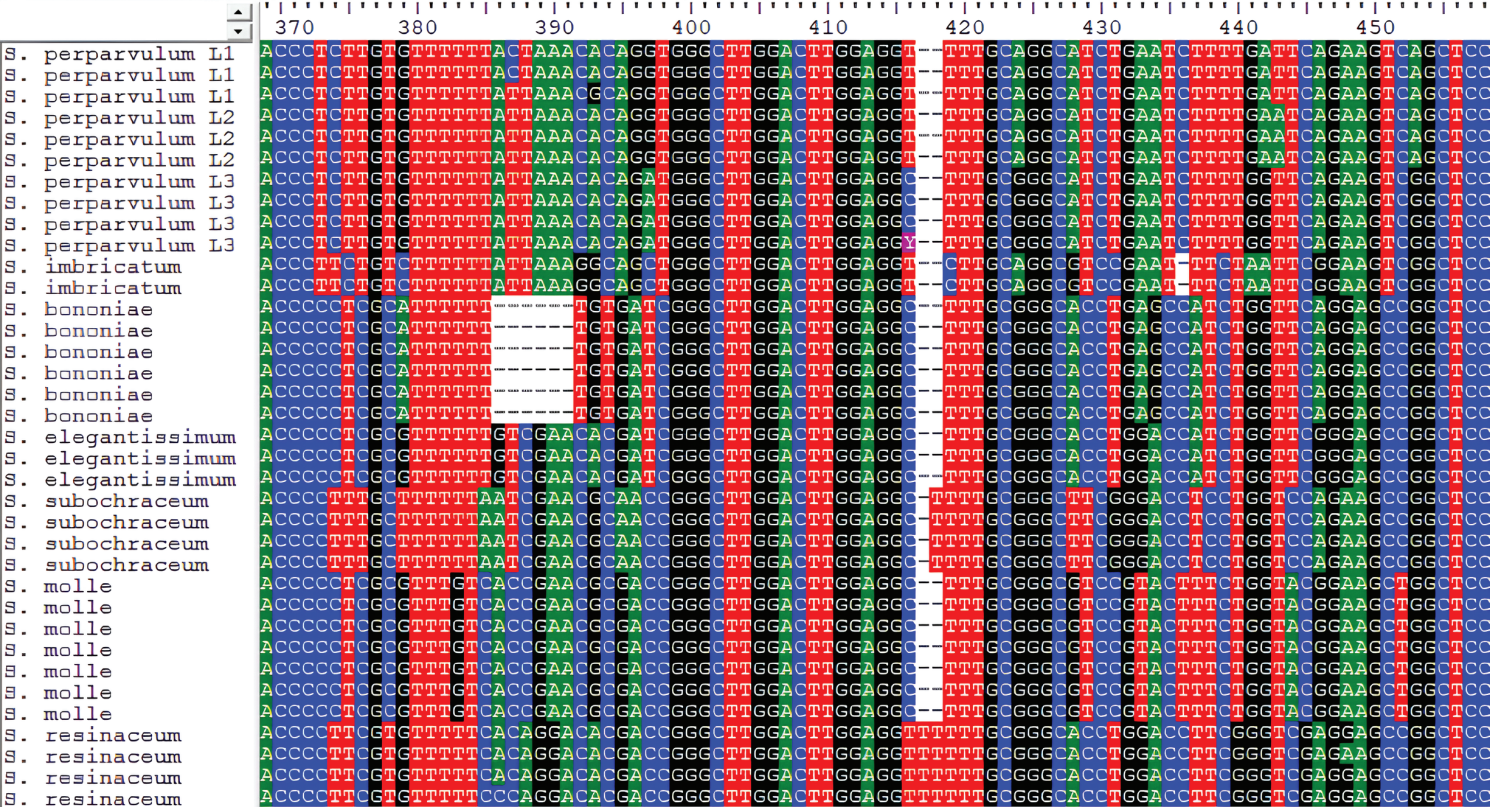
ITS2 sequences in *Steccherinum* spp. showing interspecific and intraspecific variation.

This species is primarily distinguished by having the smallest basidiospores (2.3–3.1 × 1.7–2.2 µm) of all Neotropical hydnoid/odontioid *Steccherinum*, usually measuring under 3 µm long (Table 5). Macroscopically, it typically forms large basidiomes with short aculei and a papery consistency when dried. While most of the specimens examined were strictly resupinate, some exhibited small pilei along the margins. We examined the holotype of *S. perparvulum* and found several specimens with basidiospore sizes and general morphological characteristics consistent with the species. However, our phylogenetic analyses revealed that these specimens comprise at least three distinct lineages (Figs 1, 3). These lineages show minor differences in the ITS region, although subtle variations also occur among specimens within the same lineage (Fig. 12).

To determine whether the genetic differences observed represent distinct biological species or reflect broader molecular variability within the ITS region of *S. perparvulum*, we conducted mating tests with monosporic cultures from three different specimens (Table 6). Two of these specimens belonged to the same lineage (710 and 744, lineage 1), whereas the third represented a different lineage (742, lineage 3). The mating tests showed positive results only between specimens of the same lineage, with clamp connections present in 11 of 12 monosporic pairings. In contrast, all confrontations with specimen 742 were negative, with only simple-septate hyphae present. This further supports the hypothesis that the observed molecular differences represent separate species within the group. Unfortunately, cultures from lineage 2 were not available for compatibility testing. Further studies incorporating additional monosporic cultures would be valuable to explore potential intercompatibility within the group, especially considering that lineage 2 is phylogenetically very close to lineage 1.

**Table 6.** Mating tests in the *Steccherinum perparvulum* species complex.

Monosporic culture n.	*710.1*	*710.2*	*710.3*	*742.1*	*742.2*	*742.3*
*744.1*	+	+	-	-	-	-
*744.2*	+	+	+	-	-	-
*744.3*	+	+	+	-	-	-
*744.4*	+	+	+	-	-	-
*742.1*	-	-	-			
*742.2*	-	-	-			
*742.3*	-	-	-			

While our studies corroborate at least two different species within *S. perparvulum*, no morphological or biogeographical evidence was found to support their segregation. Therefore, we chose to retain these taxa under the same name for the time being, treating it as a species complex. Considering their morphological and phylogenetic affinities, this approach aims to facilitate the taxonomy of the group rather than complicating it by increasing the number of species and adding morphologically indistinguishable taxa. In addition, at present, it is not possible to define which of the lineages represents *S. perparvulum* s.s. Sequences from the type specimen or the type locality could further elucidate this issue and help clarify the taxonomy of this species complex.

#### Steccherinum resinaceum

Taxon classificationAnimaliaPolyporalesSteccherinaceae

Westphalen & Minosso sp. nov.

589A4DB4-6D95-5DF6-AA25-9B7FAD240669

858855

Figs 8E, 9E, 11G

##### Etymology.

Refers to the hard and waxy consistency of the basidiomes when dried.

##### Diagnosis.

Distinguished from other species in the genus mainly by the waxy and dense basidiomes upon drying, the laterally fused aculei that bifurcate at apices, and the skeletocystidia encrusted with large crystals, usually embedded in the trama.

##### Typification.

Brazil. São Paulo: São Luís do Paraitinga, Parque Estadual da Serra do Mar, Núcleo Santa Virgínia, Trilha Pirapitinga, 05 Jun. 2017, M.C. Westphalen 551/17 (SP 512670).

##### Description.

Basidiomes adnate, resupinate, easily separable from the substratum and usually detaching upon drying, membranaceous to papery and pliable when fresh, turning waxy and somewhat rigid upon drying; sterile margins entire, smooth, pelliculose, up to 1 mm wide. Hymenophore hydnoid, cream to pale yellowish when fresh, turning beige to ochraceous upon drying, aculei (0.3–)0.4–0.75 × 0.15–0.3(–0.5) mm, usually with straight to bifurcate apices with a pilose appearance from the protruding cystidia, solitary or more commonly laterally fused, somewhat crowded, 5–6 per mm. Subiculum cream to beige, homogeneous, dense, up to 0.3 mm thick.

Hyphal system dimitic, subicular hyphae very compact, tramal hyphae intertwined, subparallel; generative hyphae clamped, thin to slightly thick-walled, hyaline, occasionally branched, 2–4 µm wide, more abundant in the base of the subiculum; skeletal hyphae thick-walled to almost solid, hyaline to slightly yellowish, 2–4.5 µm wide. Skeletocystidia abundant, immersed in the trama or protruding into the hymenium, coarsely encrusted with large crystals, 25–45(–50) × (7–)9–11(–12) µm, some thinner and longer cystidia also observed immersed in the subiculum, up to 60 µm long and 6–8 µm wide; leptocystidia present, abundant in the apices of the aculei, clavate, ventricose, or capitate, smooth or with a crown of crystals. Basidia clavate, tetrasterigmate, 10–12 × 3.5–5 µm. Basidiospores broadly ellipsoid, hyaline, thin-walled IKI–, CB–, 2.7–3.4(–3.5) × 1.7–2.4 µm.

##### Habitat and distribution.

Known only from Atlantic Rainforest areas in southeastern Brazil.

##### Specimens examined.

Brazil • São Paulo: São Paulo, Parque Estadual Cantareira, Núcleo Engordador, Trilha da Cachoeira, 24 Apr. 2017, M.C. Westphalen 540/17 (SP 512671); • São Luís do Paraitinga, Parque Estadual da Serra do Mar, Núcleo Santa Virgínia, Trilha Olho d’ água, 13 Feb. 2019 M.C. Westphalen 665/19 (SP 512687, ICN 213880); • São Paulo, Parque Estadual das Fontes do Ipiranga, 18 Feb. 2019, M.C. Westphalen 679/19 (SP 512669).

##### Notes.

*Steccherinum resinaceum* is characterized by its waxy and somewhat hard basidiomes when dried, a hymenophore composed of laterally fused, often bifurcating aculei, and coarsely encrusted cystidia with large crystals. Macroscopically, the basidiomes of *S. bononiae* are somewhat similar but differ in having solitary aculei with a pilose appearance due to prominently protruding skeletocystidia, whereas in *S. resinaceum* the skeletocystidia are visible only at the apices of the aculei (Fig. 8E). Additionally, the cystidia in *S. bononiae* are longer and typically covered with small- to medium-sized crystals.

*Steccherinum molle* shares similar cystidial morphology and basidiospore size with *S. resinaceum*. Nonetheless, it can be distinguished by its softer basidiomes with a cottony subiculum and slightly shorter and thinner aculei. Unfortunately, we could not obtain monosporic cultures of *S. resinaceum* to confirm its mating system.

#### Steccherinum subochraceum

Taxon classificationAnimaliaPolyporalesSteccherinaceae

Bononi & Hjortstam

E4650FA5-EAB7-5498-B996-6BF1F05720C9

Figs 8F, 9F, 10A, 11H

##### Description.

Full description in Hjortstam and Bononi (1986).

##### Mating system.

Tetrapolar. Monosporic cultures obtained from specimen 730/22.

##### Habitat and distribution.

Known from the Atlantic Rainforest in southern and southeastern Brazil.

##### Specimens examined.

Brazil • Rio Grande do Sul: São Salvador, Montenegro, 04 Apr. 1945, Rick s.n. (PACA 22824 - holotype of *Iprex hydenus* Rick); • São Francisco de Paula, FLONA, 12 Mar. 2022, M.C. Westphalen 730/22 (ICN 213881); • ibid., 25 Nov. 2023, M.C. Westphalen 748/23 (ICN 213883); • Farroupilha, Parque dos Pinheiros, 22 Jul. 2023, M.C. Westphalen 746/23 (ICN 213882); • Canela, FLONA, 13 Jul. 2024, Westphalen 761/24 (ICN 313884). • São Paulo: São Paulo, Parque Estadual das Fontes do Ipiranga, 06 Oct. 1966, H. Requejo s.n. (SP 97591 - holotype).

##### Notes.

*S. subochraceum* was described from southeastern Brazil to provide a valid name for *Iprex hydenus*, originally described by Rick (1959) but considered invalid due to the lack of a designated type specimen. Among Neotropical hydnoid *Steccherinum* species, *S. subochraceum* is readily distinguished by the effused-reflexed basidiomes with notably long aculei (Figs 8F, 10A), usually measuring over 1.5 mm long (0.85–2.25 × 0.15–0.45 mm) (Table 4). Microscopically, it presents thin, elongated cystidia [25–60(–70) × 5–8(–10) µm] immersed in the trama, sometimes protruding at the apices of the aculei, and subglobose basidiospores measuring (3.2–)3.3–4.2 × 2.5–3.8 µm. *Steccherinum larssonii* and *Steccherinum basibadium* share a similar basidiospore size range (3.5–4.5 × 3.0–3.5 µm and 3.6–4.5 × 3.1–3.4 µm, respectively) but can be distinguished by their significantly shorter aculei, which reach a maximum length of 0.75 mm. In addition, *Steccherinum basibadium* forms more developed pilei with a brownish surface, whereas *S. larssonii* often produces completely resupinate basidiomes or only small pilei at the margins (Maas Geesteranus 1974; Westphalen et al. 2021).

The subicular generative hyphae of *S. subochraceum* are regularly clamped, although simple-septate hyphae were observed in the trama of some specimens. Simple-septate hyphae were readily observed in specimen 746/23 but were less common in 730/22. Phylogenetically, *S. subochraceum* forms a sister clade to *S. undigerum*, a Neotropical species with somewhat similar basidiome morphology (effused-reflexed with imbricate pilei) and that also exhibits a strongly fimbriate mycelium in culture. However, *S. undigerum* differs in having a poroid hymenophore with dentate dissepiments and slightly larger basidiospores (4–5 × 3.5–4.5 μm).

#### Steccherinum undulatum

Taxon classificationAnimaliaPolyporalesSteccherinaceae

Westphalen & R.M. Silveira sp. nov.

82022089-43AC-52D2-8067-11C6C4DAE67C

858853

Figs 8G, 9G, 10B, 11I

##### Etymology.

Refers to the basidiomes with small, wavy pilei.

##### Diagnosis.

Recognized mainly by the combination of basidiomes formed by several small, wavy, imbricate pilei, aculei with 0.75–1 mm long, and ellipsoid basidiospores 3.3–4.1 × 2.0–2.7(–2.8) µm.

##### Typification.

Brazil. Rio Grande do Sul: São Francisco de Paula, CPCN Pró-Mata, 20 Apr. 2023, M. C. Westphalen 743/23 (ICN 213872).

##### Description.

Basidiomes adnate, concrescent, effused-reflexed, with several small, wavy, imbricate pilei, formed by the coalescence of several small pilei with conjoined reflexed bases, usually not detaching when dried, but easily separable if pulled from the substratum, somewhat membranaceous and pliable when fresh, turning papery to corky upon drying and somewhat waxy; pilei small, up to 4 mm wide and 9 mm in length, pilear surface cream to beige, tomentose, sulcate, and sometimes faintly zonate; sterile margins entire, somewhat cottony, smooth to finely fimbriate, up to 1.5 mm wide. Hymenophore hydnoid, at first orange in young and fresh specimens, then turning pale salmon to beige with age and upon drying, aculei 0.75–1.0 × 0.2–0.4 mm, with acute to round apices, sometimes slightly asperulate from the protruding cystidia, solitary or more rarely laterally fused, crowded, 4–6 per mm. Subiculum white, homogeneous, up to 0.6 mm thick.

Hyphal system dimitic, subicular hyphae compact, not agglutinated, tramal hyphae parallel, intertwined, and densely arranged; generative hyphae clamped, thin to slightly thick-walled, hyaline, occasionally branched, 2–4 µm wide; skeletal hyphae thick-walled to almost solid, abundant throughout the basidiome, hyaline, 2–5 µm wide. Skeletocystidia abundant, immersed in the trama or with the apices protruding into the hymenium, covered with a thick cap of small crystals, 20–60 × 7–10 µm; leptocystidia present, often scattered and somewhat inconspicuous, mostly clavate or with a rounded apex, smooth. Basidia clavate, tetrasterigmate, 12–16 × 4–5 µm. Basidiospores ellipsoid, hyaline, thin-walled, IKI-, CB-, 3.3–4.1 × 2.0–2.7(–2.8) µm.

##### Habitat and distribution.

Known only from Araucaria forests in southern Brazil.

##### Specimens examined.

Brazil • Rio Grande do Sul: Canela, FLONA, 13 Jul. 2024, M.C. Westphalen 760/24 (ICN 213873).

##### Notes.

This species can be distinguished by its basidiomes consisting of small, wavy, imbricate pilei (Fig. 10B). One of the collections studied (760/24) exhibited a bright orange coloration when fresh, whereas the other (743/23) displayed a more faded salmon hue. This suggests that only young, fresh specimens display brighter colors, which gradually turn paler as they mature or dry. The aculei of *S. undulatum* are the second largest in the group, being smaller only than those of *S. subochraceum*. Microscopically, the species also features slightly larger basidiospores when compared to *S. molle*, *S. resinaceum*, and *S. bononiae*, but smaller than those of *S. elegantissimum*.

Although surveys for *Steccherinum* species have been ongoing since 2017, only two specimens of *S. undulatum* have been found, both in Araucaria forests in southern Brazil, approximately 45 km apart. This suggests that it is likely rarer than other species in the group, which have been more frequently collected and are more widely distributed.

### Identification key to Brazilian corticioid species of *Steccherinum* and *Cabalodontia*

**Table:** 

1	Basidiomes soft and brittle, hyphal system monomitic to pseudo-dimitic, cystidia tapering towards the apex or more rarely clavate	**2 (*Cabalodontia* )**
–	Basidiomes membranaceous to waxy, hyphal system dimitic, cystidia clavate	**4 (*Steccherinum* )**
2	Hymenophore in shades of brown, farinaceous, aculei cluttered and somewhat indistinct, hyphae golden yellow in mass	***C. brunnea***
–	Hymenophore white to beige, not farinaceous, hyphae hyaline, aculei distinct	**3**
3	Aculei up to 0.4 mm long, basidiospores subglobose to oblong-ellipsoid, 3.5–4(–4.5) × 2.5–3(–3.5) μm	***C. delicata***
–	Aculei longer, up to 0.8 mm, basidiospores ellipsoid to narrowly-ellipsoid, (4–)4.3–5.1(–5.3) × 2.4–3(–3.1) μm	***C. albofulva***
4	Aculei long, conspicuous, readily visible to the naked eye, mostly ≥ 1.5 mm	***S. subochraceum***
–	Aculei shorter, inconspicuous, or not readily visible to the naked eye, ≤ 1.0 mm.	**5**
5	Basidiomes effused-reflexed, pilei small, imbricate, forming a wavy pattern, aculei 0.75–1.0 mm long (Lm = 0.85 mm)	***S. undulatum***
–	Basidiomes resupinate to effused-reflexed, pilei absent or marginal, not in a wavy pattern, aculei up to 0.75 mm long (Lm < 0.7 mm)	**6**
6	Basidiospores 3.5–5.0 × 2.5–4.0 μm	**7**
–	Basidiospores smaller, 2.3–3.5 × 1.7–2.5 μm	**8**
7	Basidiospores subglobose, 3.5–4.3 × 3–3.7 μm; cystidia 7–10 µm wide	***S. larssonii***
–	Basidiospores ellipsoid, 3.5–5.0 × 2.2–2.9 µm; cystidia 4–6(8) µm wide	***S. elegantissimum***
8	Cystidia coarsely encrusted with chunky crystals, embedded in the trama or slightly protruding into the hymenium	**9**
–	Cystidia evenly covered with thin to medium-sized crystals, mostly projecting above the hymenium	**10**
9	Basidiomes waxy, aculei often bifurcate or laterally fused, subiculum dense	***S. resinaceum***
–	Basidiomes soft, aculei not bifurcate or laterally fused, subiculum cottony	***S. molle***
10	Basidiospores broadly ellipsoid 2.3–3.0(3.2) × 1.7–2.2 µm	***S. perparvulum***
–	Basidiospores ellipsoid 2.6–3.6 × 1.8–2.5 µm	***S. bononiae***

## Discussion

Through the integration of morphological, molecular, and culture data, this study expands the knowledge of *Steccherinum* in the Neotropics by describing five new species and providing newly obtained molecular data on *S. perparvulum* and *S. subochraceum*. Along with *S. larssonii* and *S. basibadium*, these seven species share many morphological traits and can only be reliably distinguished through a combination of characteristics, such as aculei size, basidiospore size and shape, and the incrustation and position of cystidia. However, considerable overlap in these features among taxa makes species identification challenging without a thorough comparison of multiple specimens. Furthermore, molecular data revealed that *S. perparvulum* represents a species complex comprising at least three distinct lineages that are morphologically indistinguishable and exhibit no clear differences in distribution, with some lineages occurring sympatrically. In addition to the new data on *Steccherinum*, two new species and two new combinations are proposed in *Cabalodontia*, doubling the number of known taxa in the genus.

### Morphology and ecology of *Cabalodontia* spp.

The genus *Cabalodontia* differs from *Steccherinum* mainly by its fragile and brittle basidiomes, a monomitic hyphal system, and cystidia tapering toward the apex. However, some clavate cystidia were also observed in the analyzed specimens, resembling those found in *Steccherinum* s.s. In addition, two taxa recovered within *Cabalodontia*, *C. tenuissima* and *Irpex oreophilus*, have been described as dimitic. The placement of *I. oreophilus* within *Cabalodontia* was discussed by Westphalen et al. (2021), who noted that the absence of sequence data from the type specimen or type locality prevents confirmation of its generic placement. Therefore, a conservative approach is followed here, and no new combination is proposed for this species at present.

Interpretation of the hyphal system in *Cabalodontia* may be challenging in some cases due to the presence of thick-walled hyphae that give rise to cystidia. While some authors may interpret these segments as skeletal hyphae, all examined *Cabalodontia* specimens show abundant generative hyphae with clamp connections. This contrasts with *Steccherinum*, where the hyphal system is clearly dimitic, with abundant skeletal hyphae. A similar pattern is evident in the original description of *C. tenuissima* (Wu et al. 2021a), in which the line drawings depict predominantly clamped hyphae and only a few sclerified hyphae in the subiculum. Type studies of *I. oreophilus*, in turn, revealed more sclerified hyphae compared to other species in the group; however, thick-walled clamped hyphae and abundant generative hyphae were also observed. For these reasons, the hyphal system in *Cabalodontia* is best interpreted as ranging from monomitic to pseudo-dimitic, which also helps distinguish the genus from truly dimitic taxa in *Steccherinum* and *Junghuhnia*. If future studies confirm truly dimitic taxa within the genus, its circumscription would need to be expanded.

The three Brazilian species of *Cabalodontia* currently known (*C. albofulva*, *C. brunnea*, and *C. delicata*) were collected in high-altitude areas above 800 m, mostly in Araucaria forests. This distribution suggests a preference for subtropical conditions in the Neotropics, particularly given the absence of records from northern Brazil. While *C. delicata* is relatively frequent in high-altitude forests, *C. brunnea* and *C. albofulva* appear to be rare and are each known from a single collection, despite several field expeditions conducted in nearby regions over the past seven years.

### Morphology and ecology of *Steccherinum* spp.

The hymenophore morphology is a key feature to distinguish taxa in *Steccherinum* because of the variation in size and number of aculei per millimeter. In addition, the aculei may be solitary or fused, their apices acute, straight, rounded, or bifurcate, and pilose or not due to protruding skeletocystidia. These features, along with the general basidiome consistency when dried and the arrangement of the subicular hyphae, should be carefully examined for species identification. Although basidiospores are often of significant importance in differentiating closely related taxa, among the five new species described, only *S. elegantissimum* exhibits distinctly larger spores, yet still presents some overlap, especially with *S. undulatum*. All the remaining species have very small and similar basidiospores, which do not represent a reliable taxonomic feature to distinguish them. Nevertheless, basidiospore size and shape remain important features to consider when examining Neotropical *Steccherinum* spp., particularly when assessed alongside other morphological characteristics.

Regarding the cystidia, two main types of incrustations have been observed in *Steccherinum* spp.: (1) skeletocystidia covered with small to medium crystals and (2) coarsely encrusted cystidia with large crystals (Fig. 11K, L). In addition, while in some species the cystidia are long and project above the hymenium, in others they are embedded in the trama or have only slightly projecting apices. In contrast, cystidia in *Cabalodontia* are typically widened at the base, tapering toward the apex, and encrusted with large crystals (Fig. 11J). It is important to note, however, that variation in cystidial incrustation, shape, and position may occur in some cases, and these features should be considered as additional characteristics when assessing their overall appearance across different species, which can help differentiate them.

Regarding the ecology of the studied taxa, most *Steccherinum* species were found growing on fallen branches of angiosperms, suggesting that they occupy similar ecological niches and perform comparable ecological roles. The only exception was *S. larssonii*, which was collected both from fallen branches and logs of unidentified angiosperms. In addition, no clear biogeographic pattern was observed, as several species occurred within the same or nearby regions. Only *S. larssonii*, *S. resinaceum*, and *S. undulatum* exhibited more restricted distributions, with *S. larssonii* and *S. resinaceum* being found exclusively in southeastern Brazil and *S. undulatum* in montane Araucaria forests in the southern region. Nevertheless, it is highly probable that all of the species treated in this study are widespread throughout the Atlantic Rainforest biome, with some, such as *S. undulatum*, potentially restricted to montane environments. Broader sampling across different forest types and elevations would help clarify species ranges and their ecological preferences.

### Culture studies data

The culture studies revealed distinct patterns among the analyzed taxa. While *S. bononiae* and *S. elegantissimum* exhibited slower growth rates, *S. perparvulum* and *S. resinaceum* showed comparatively faster growth. At the same time, notable intraspecific variation was observed in the growth rates of *S. undulatum* and *S. subochraceum*, highlighting variability within species. Further comparisons were made between cultures from two of the three *S. perparvulum* lineages; however, no significant differences in growth rates or morphological traits were detected (Table 3, Fig. 6). These findings suggest that, although growth patterns and mycelial macromorphology can be useful for taxonomic identification in *Steccherinum*, intraspecific variation and overlapping characteristics must be considered. To achieve more accurate results, broader sampling of cultures is necessary. Expanding the dataset could help identify more consistent patterns and refine criteria that can be used to aid in distinguishing taxa.

Through monosporic confrontations, we were able to test the mating system of five of the *Steccherinum* species studied (*S. bononiae*, *S. elegantissimum*, *S. molle*, *S. perparvulum*, and *S. subochraceum*), confirming that all are tetrapolar, a characteristic also seen in other species of the genus (Rajchenberg 2011; Westphalen et al. 2018). Additional mating tests were also conducted among monosporic cultures of the different taxa to assess potential sexual compatibility. All matings were negative, confirming that the taxa are reproductively isolated and represent distinct biological species.

### Molecular data and divergence time estimates

Comparative analysis of the ITS sequences among the studied species revealed distinct regions, particularly at the beginning of ITS2, that are unique and can be used to reliably differentiate taxa within the group (Fig. 12). Although some variation was also detected in the ITS region of the *S. perparvulum* complex, it is less pronounced than the differences observed among the other species. For example, in the highlighted ITS2 region, variation is limited to approximately seven scattered base changes. In contrast, comparison between the sister species *S. elegantissimum* and *S. bononiae* reveals 13 base substitutions, comprising longer segments with more pronounced differences (Fig. 12). It is noteworthy that, in spite of the genetic variation observed in ITS as well as *tef*1-α, the species within the group exhibit a high degree of morphological similarity. This pattern could suggest that speciation in these taxa may be relatively recent, particularly given their shared ecological niches and overlapping distributions. Regarding the *S. perparvulum* clade, the three lineages found are estimated to have been segregated for at least 10.39 Myr (95% height = 15.73–5.46 Myr), indicating that morphological differentiation between taxa can take more than 10 million years to occur. To elucidate this, the group would benefit from further analyses encompassing the biogeographic history and diversification rates of its taxa.

Recently, Deng et al. (2025) provided the first analysis of divergence time estimates of the *Steccherinaceae*, encompassing 16 different genera. In turn, our study provides more comprehensive sampling with 19 genera and several new sequences of Neotropical taxa, especially of *Steccherinum* and *Cabalodontia*. The five-gene dataset utilized to obtain the MCC tree (Fig. 3) represents the most comprehensive phylogram constructed for the family to date, excluding only monotypic or poorly sequenced genera from the analysis. In this sense, the low support obtained in some of the nodes that form the backbone of *Steccherinaceae* likely reflects the scarcity of molecular data and the existence of yet undescribed taxa within the family. It is noteworthy that the same clades tend to shift according to the dataset applied, something that may only be resolved through increased sampling and phylogenomic analyses of the group.

Regarding the molecular dating, our analysis retrieved *Steccherinaceae* with a mean crown age of 86.4 Myr, with an HPD value that is consistent with the 113 Myr estimation obtained by He et al. (2024) through maximum likelihood analysis of phylogenomic data. Although slightly older, it also corroborates the mean crown age of 120.8 Myr obtained by Deng et al. (2025), placing the origin of the family in the early to late Cretaceous period. The differences between the values are probably the result of differences in the datasets and genes selected or the slightly different methodologies applied, especially the inclusion of a long-distance fossil calibration point representing the segregation of Ascomycota and Basidiomycota (Deng et al. 2025). In addition, the estimation indicates that most taxa within *Steccherinaceae* have emerged in the Cenozoic Era, between the Paleocene and Oligocene, with an overall younger age compared to genera of other families in *Polyporales*, such as *Irpicaceae*, *Meripilaceae*, and *Meruliaceae* (Wang et al. 2022; Li et al. 2025a; Wang et al. 2025). This more recent speciation event could explain the species complexes and morphological similarities shared among related taxa, especially in hydnoid-poroid genera such as *Steccherinum*, *Cabalodontia*, and *Junghuhnia*.

Considering that the majority of the known species in *Steccherinaceae* are wood decomposers, their evolutionary history likely follows the same path as taxa within the previously mentioned families, tracking the angiosperm radiation and expansion that started in the Late Cretaceous and presented a diversification surge in the Cenozoic Era, marked by global climatic cooling that could explain this shift (Varga et al. 2019; Zuntini et al. 2024). Nevertheless, the Neotropical distribution and speciation of the family, especially for *Steccherinum* spp., could be intrinsically related to the biogeographic history of the Atlantic Rainforest, from which most of the known species were described. In this sense, the Araucaria forests, present in the southern portion of the biome for at least 200 Myr, possess a complex biogeographic and evolutionary history that could be linked to the speciation processes observed in the group (Vasconcellos et al. 2024).

Although the divergence estimates presented here provide valuable insights into the evolutionary history of the group, the estimated ages of most genera should be interpreted with caution until additional molecular data allow for more comprehensive analyses encompassing the genetic diversity of *Steccherinaceae* and its taxa. Therefore, continued efforts to expand the molecular and phylogenomic data of the group will be essential to clarify the evolutionary relationships among its genera.

### Additional neotropical *Steccherinum* s.l. taxa

In addition to the species discussed in this study, two other odontioid/hydnoid *Steccherinum* species have been described in the Neotropics: *S. filiferum* (Yurchenko et al. 2023) and *S. diversum* (Hjortstam 1999). We examined images of the *S. diversum* type specimen, kindly sent by the Kew Herbarium staff, and found that it differs from all the studied species by presenting basidiomes that are strongly attached to the substrate, a loose subiculum, and brittle aculei. These morphological traits deviate from typical *Steccherinum* s.s. This is particularly relevant given that several species originally described in *Steccherinum* have been shown, through phylogenetic analyses, to belong to other genera (Miettinen et al. 2012; Miettinen and Ryvarden 2016; Westphalen et al. 2021). Furthermore, in its original description, Hjortstam (1999) considered *S. diversum* morphologically similar to *S. laeticolor* (Berk. & M.A. Curtis) Banker, a species that is phylogenetically related to *Junghuhnia* rather than *Steccherinum*. Future studies incorporating new collections and molecular data may help determine if *S. diversum* should remain in *Steccherinum* or be transferred to another genus. Unfortunately, we were unable to examine specimens of *S. filiferum*. However, the species can be readily distinguished from other Neotropical odontioid *Steccherinum* by its fragile basidiomes, monomitic hyphal system, and simple-septate generative hyphae. Phylogenetically, it nests in a clade alongside *S. amapaense*, *S. fragile*, an*d S. laxum* (Fig. 1), all of which share somewhat soft or fragile basidiomes, a monomitic to pseudo-dimitic hyphal system, and simple-septate generative hyphae. These morphological traits deviate from *Steccherinum* s.s., which is characterized by a dimitic hyphal system and clamped generative hyphae. This suggests that these taxa could be transferred to a new genus, provided that no existing older name is available for the group. However, *S. rubigimaculatum* also presents simple-septate generative hyphae but differs by having a dimitic hyphal system, and it falls into a separate, distantly related lineage. As previously mentioned, many of the outer clades in our molecular analysis showed low support, especially regarding the basal groups in *Steccherinum* s.l. Interestingly, *S. tenue* nested near the simple-septate/monomitic species but with low support, despite possessing a dimitic hyphal system and clamped generative hyphae. Additionally, previous studies (Miettinen et al. 2012; Westphalen et al. 2021) have placed *S. tenue* in different clades within *Steccherinum*, suggesting that the currently available molecular data are insufficient to resolve its true phylogenetic position or to strongly support the transfer of *S. amapaense*, *S. fragile*, *S. filiferum*, and *S. laxum* to a different genus.

In conclusion, this study represents a significant advancement in the understanding of the genus *Steccherinum*, providing precise morphological data, the addition of over 110 new DNA sequences from five distinct regions, mating system determination of five species, mycelial culture data, and divergence time estimates. However, it is important to highlight that several recently described species assigned to *Steccherinum* are not phylogenetically nested within the genus (unpublished data), requiring further studies to determine their correct generic placement. Additionally, other taxa not included in the present study due to insufficient morphological or sequence data may represent additional species within the genus in the Neotropical region, underscoring the need for continued research on the *Steccherinaceae* to accurately assess the group’s true diversity in the region.

## Supplementary Material

XML Treatment for Cabalodontia albofulva

XML Treatment for Cabalodontia brunnea

XML Treatment for Cabalodontia lincangense

XML Treatment for Cabalodontia tenuissima

XML Treatment for Steccherinum bononiae

XML Treatment for Steccherinum elegantissimum

XML Treatment for Steccherinum molle

XML Treatment for Steccherinum perparvulum

XML Treatment for Steccherinum resinaceum

XML Treatment for Steccherinum subochraceum

XML Treatment for Steccherinum undulatum

## Additional information

### Use of AI

No use of AI was reported.

### Adherence to national and international regulations

All the fungal strains used in this study have been legally obtained, respecting the Convention on Biological Diversity (Rio Convention).

### Author ORCIDs

Mauro Carpes Westphalen https://orcid.org/0000-0001-5346-3541

Nathalia Michele Martins Minosso https://orcid.org/0009-0005-1080-5392

Nicolas do Carmo Regio https://orcid.org/0000-0002-2778-5384

Adriana de Mello Gugliotta https://orcid.org/0000-0002-0241-7825

Mario Rajchenberg https://orcid.org/0000-0001-5031-5148

Rosa Mara Borges da Silveira https://orcid.org/0000-0003-1578-5034

### Data availability

All of the data that support the findings of this study are available in the main text or Supplementary Information.

## Supplementary materials

Supplementary material 1 Alignment *Steccherinum* Data type fas Explanation note Alignment used for phylogenetic analysis of the genus *Steccherinum* (Fig. 1). https://binary.pensoft.net/file/1550427This dataset is made available under the Open Database License (http://opendatacommons.org/licenses/odbl/1.0/). The Open Database License (ODbL) is a license agreement intended to allow users to freely share, modify, and use this Dataset while maintaining this same freedom for others, provided that the original source and author(s) are credited. Mauro Carpes Westphalen, Nathalia Michele Martins Minosso, Nicolas do Carmo Regio, Adriana de Mello Gugliotta, Mario Rajchenberg, Rosa Mara Borges da Silveira

Supplementary material 2 Alignment C*abalodontia* Data type fas Explanation note Alignment used for phylogenetic analysis of the genus C*abalodontia* (Fig. 2). https://binary.pensoft.net/file/1550428This dataset is made available under the Open Database License (http://opendatacommons.org/licenses/odbl/1.0/). The Open Database License (ODbL) is a license agreement intended to allow users to freely share, modify, and use this Dataset while maintaining this same freedom for others, provided that the original source and author(s) are credited. Mauro Carpes Westphalen, Nathalia Michele Martins Minosso, Nicolas do Carmo Regio, Adriana de Mello Gugliotta, Mario Rajchenberg, Rosa Mara Borges da Silveira

Supplementary material 3 Alignment - *Steccherinaceae* divergence times Data type fas Explanation note Alignment used for the divergence time estimates phylogenetic tree (Fig. 3). https://binary.pensoft.net/file/1550429This dataset is made available under the Open Database License (http://opendatacommons.org/licenses/odbl/1.0/). The Open Database License (ODbL) is a license agreement intended to allow users to freely share, modify, and use this Dataset while maintaining this same freedom for others, provided that the original source and author(s) are credited. Mauro Carpes Westphalen, Nathalia Michele Martins Minosso, Nicolas do Carmo Regio, Adriana de Mello Gugliotta, Mario Rajchenberg, Rosa Mara Borges da Silveira
